# System biology approach to identify the novel biomarkers in glioblastoma multiforme tumors by using computational analysis

**DOI:** 10.3389/fphar.2024.1364138

**Published:** 2024-05-22

**Authors:** Safar M. Alqahtani, Ali Altharawi, Alhumaidi Alabbas, Faisal Ahmad, Hassan Ayaz, Asia Nawaz, Sidra Rahman, Manal A. Alossaimi

**Affiliations:** ^1^ Department of Pharmaceutical Chemistry, College of Pharmacy, Prince Sattam Bin Abdulaziz University, Al Kharj, Saudi Arabia; ^2^ Foundation University Medical College, Foundation University Islamabad, Islamabad, Pakistan; ^3^ School of Biology Georgia Institute of Technology, Atlanta, GA, United States; ^4^ Department of Biotechnology, Quaid-i-Azam University Islamabad, Islamabad, Pakistan

**Keywords:** biomarker, gene expression, glioblastoma, protein–protein interactions, molecular dynamic simulations

## Abstract

**Introduction:** The most common primary brain tumor in adults is glioblastoma multiforme (GBM), accounting for 45.2% of all cases. The characteristics of GBM, a highly aggressive brain tumor, include rapid cell division and a propensity for necrosis. Regretfully, the prognosis is extremely poor, with only 5.5% of patients surviving after diagnosis.

**Methodology:** To eradicate these kinds of complicated diseases, significant focus is placed on developing more effective drugs and pinpointing precise pharmacological targets. Finding appropriate biomarkers for drug discovery entails considering a variety of factors, including illness states, gene expression levels, and interactions between proteins. Using statistical techniques like p-values and false discovery rates, we identified differentially expressed genes (DEGs) as the first step in our research for identifying promising biomarkers in GBM. Of the 132 genes, 13 showed upregulation, and only 29 showed unique downregulation. No statistically significant changes in the expression of the remaining genes were observed.

**Results:** Matrix metallopeptidase 9 (MMP9) had the greatest degree in the hub biomarker gene identification, followed by (periostin (POSTN) at 11 and Hes family BHLH transcription factor 5 (HES5) at 9. The significance of the identification of each hub biomarker gene in the initiation and advancement of glioblastoma multiforme was brought to light by the survival analysis. Many of these genes participate in signaling networks and function in extracellular areas, as demonstrated by the enrichment analysis.We also identified the transcription factors and kinases that control proteins in the proteinprotein interactions (PPIs) of the DEGs.

**Discussion:** We discovered drugs connected to every hub biomarker. It is an appealing therapeutic target for inhibiting MMP9 involved in GBM. Molecular docking investigations indicated that the chosen complexes (carmustine, lomustine, marimastat, and temozolomide) had high binding affinities of −6.3, −7.4, −7.7, and −8.7 kcal/mol, respectively, the mean root-mean-square deviation (RMSD) value for the carmustine complex and marimastat complex was 4.2 Å and 4.9 Å, respectively, and the lomustine and temozolomide complex system showed an average RMSD of 1.2 Å and 1.6 Å, respectively. Additionally, high stability in root-mean-square fluctuation (RMSF) analysis was observed with no structural conformational changes among the atomic molecules. Thus, these in silico investigations develop a new way for experimentalists to target lethal diseases in future.

## 1 Introduction

Glioblastoma multiforme (GBM) is a major form of primary brain tumor with a high incidence in the adult population, i.e., 45.2% of all the primary brain and central nervous system tumors. It has been categorized as a grade IV cancer by the WHO. GBM is highly malignant, mitotically active, and has a predisposition to necrosis with a poor prognosis. The survival rate of GBM patients is quite low as only 5.5% of patients survive after the diagnosis (Kanderi and Gupta, 2023a). GBM is associated with many genetic and epigenetic mutations. Genome-wide studies have revealed different risk factors that can increase the chances of GBM, including low susceptibility to allergy, defective immune system, genetic factor, and some single-nucleotide polymorphisms (SNPs) (Wrensch et al., 2009).

The survival rate for older people with GBM is unlike that for individuals aged from 20 to 39 years. This is mainly due to the suboptimal treatment therapy. Several different strategies are adopted for the treatment of GBM, including radiotherapy, chemotherapy (use of alkylating drugs such as temozolomide (TMZ) and antiangiogenic drugs such as bevacizumab), and surgical interventions. Some novel treatments and chemotherapy have proven to be promising in the prognosis of GBM. However, the overall survival rate and quality of life among GBM patients remain dismal (Kumari and Kumar, 2023). The design and incorporation of more potent drugs and the precise selection of treatment strategies including drug targets can possibly optimize the survival rate in GBM patients. Multi-omics data, including genomics, transcriptomics, and proteomics, can reveal important information that can be used for the optimization of prognosis and treatment methods. In this regard, utilizing *in silico* methods to anticipate structural consequences of mutations will prove highly valuable in gaining insights into the mechanisms behind drug resistance, allowing for a quantitative assessment of the resulting phenotypic resistance outcomes.

Network-based gene expression profiling is one of the recommended tools for discovering drug targets considering different aspects, including phases of disease, severity of disease indicated by the expression of certain genes, and protein–protein interactions (PPIs) (Su et al., 2018). System biology is a holistic approach that is adopted globally for the drug design and search of novel drug targets by incorporating all the linked components including genes, proteins, and enzymes rather than considering only a single component of this complicated and interconnected mesh (Noor et al., 2023b). It has been revealed that all these related genes and proteins interact and work coherently to construct a molecular network that plays a specific role in a pathological condition (Khan et al., 2020; Alamri and Tahir ul Qamar, 2024).

To find the potential biomarkers for GBM, an integrated approach was used that included the proteomic and transcriptomic modeling of molecular networks of microarray data (Noor et al., 2023a) that have not been established previously. We used gene expression data for defining the prospective gene/protein biomarkers that can be targeted for the treatment of glioblastoma multiforme utilizing the system biology approach based on microarray datasets. For the identification of differentially expressed genes (DEGs), the statistical method *p-*value and false discovery rate (FDR) were used, followed by survival and expression analyses and the construction of subnetwork modules. The obtained data for DEGs were evaluated for functional and structural roles via KEGG pathways and the interpretation of cellular components. Hub genes were identified, in which the matrix metallopeptidase 9 (MMP9) gene was selected as a biomarker gene.

The degradation of the extracellular matrix (ECM) is a crucial physiological process facilitated by matrix metalloproteinases (MMPs), which are zinc-dependent endopeptidases (Mondal et al., 2020). Research has shown that the ECM plays a significant role in the advancement of cancer (Dobra et al., 2023). Several ECM proteins, including fibronectin, thrombospondin-1, laminin, and osteopontin, influence the biological characteristics of tumors via their impact on cell migration and angiogenesis. The interaction between cancer cells and ECM components is crucial for both cell transformation and the development of cancer (Jabłońska-Trypuć et al., 2016). MMPs play several biological roles throughout all phases of cancer, ranging from the initial stages to the formation of metastases, in addition to their role in ECM degradation (Chambers and Matrisian, 1997; Mannello et al., 2005). Although MMPs are linked to the survival and dissemination of cancer cells, they are produced by cancer cells in minimal quantity. Cancer cells induce the production of MMPs in neighboring host cells by the secretion of interleukin, interferon, growth factors, and extracellular MMP inductors, hence exhibiting a paracrine mechanism (Dobra et al., 2023). Additionally, there were reports of increased levels of MMP9 in human brain tumors throughout the 1990s (Stetler-Stevenson, 1990). Rao et al. demonstrated a notable increase in the expression of MMP9 in highly malignant gliomas, which is shown to be associated with the course of the disease. This finding implies that MMP9 may play a role in facilitating the observed invasiveness (Rao et al., 1996). Molecular docking and molecular dynamic simulation of potential drugs for targeted genes provided valuable information that can provide the basis for further research to develop a better understanding of disease mechanisms and optimization of therapeutic agents that can upscale the process of diagnosis and treatment of glioblastoma multiforme.

## 2 Materials and methods

**FIGURE 1:**
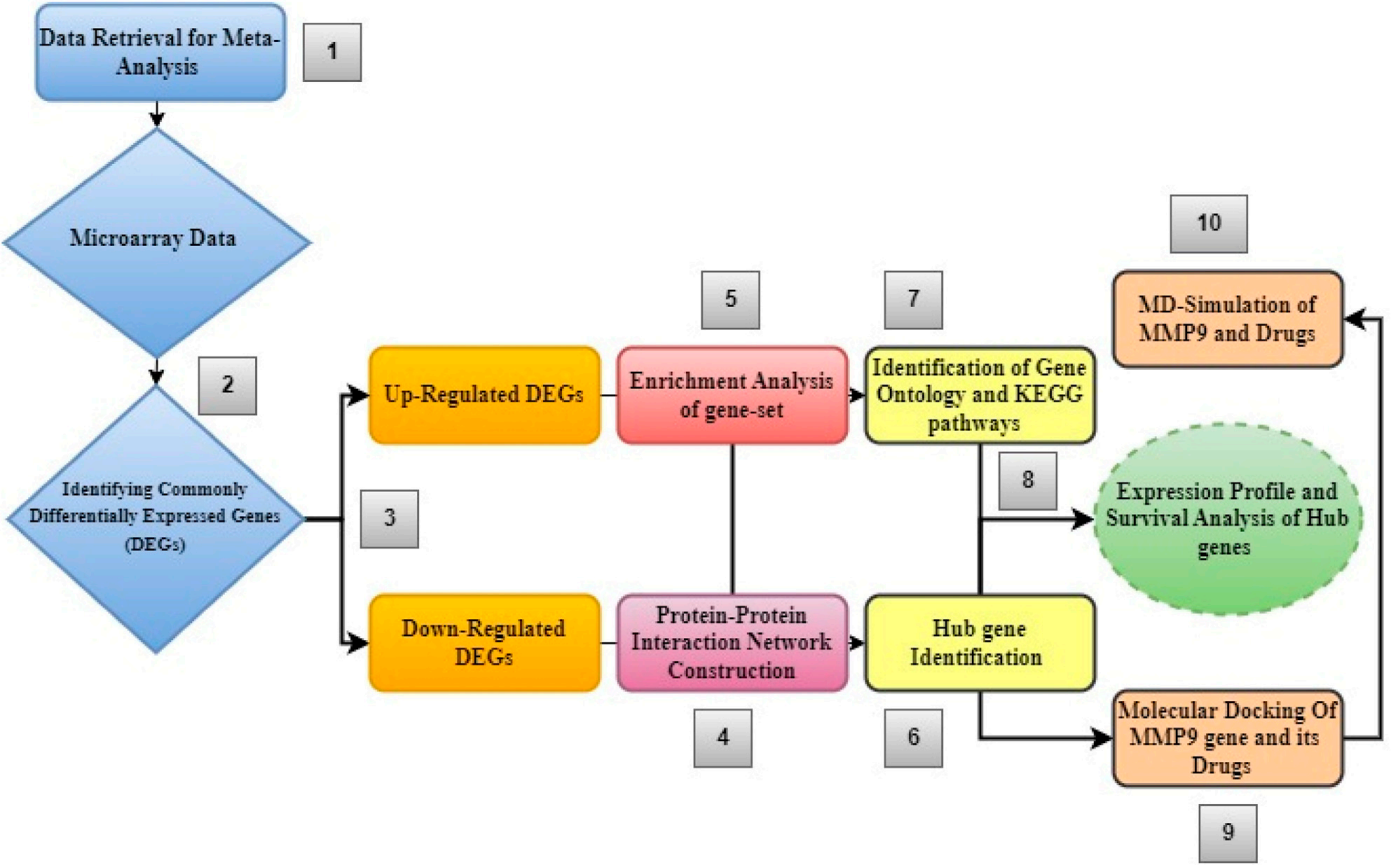
Diagram showing the order of stages of the integrated bioinformatics analytical approach used in this study.

Figure 1 shows the step-by-step process of an integrated system biology analytical technique used to find novel biomarkers and their associated pathways connected to glioblastoma multiforme tumors.

### 2.1 Retrieval dataset

The Gene Expression Omnibus (GEO) database of NCBI (https://www.ncbi.nlm.nih.gov/geo/), a freely accessible database that offers a full set of microarrays, high-throughput hybridization, chips, and other resources for research purposes, was used to collect the gene expression profile (GSE11100) of glioblastoma multiforme, which has a total of 22 samples, including from the healthy vs malignant regions of the human brain [2]. GSM280297, GSM280298, GSM280360, GSM280361, GSM280362, GSM280363, GSM280367, GSM280368, GSM280375, and GSM280376 were used as human brain healthy samples, and GSM279179, GSM279182, GSM279184, GSM279187, GSM280369, GSM280370, GSM280371, GSM280372, GSM280373, GSM280374, GSM280377, and GSM280378 were used as the human brain malignant samples. This information is based on the GPL570 Affymetrix Human Genome U133 Plus 2.0 Array (HG-U133_Plus_2). In this study, we used a variety of analytical techniques. We first investigated differential gene expression and then used principal component analysis (PCA) to organize data with related properties. To demonstrate the genes that showed differential expression visually, we also used heatmaps. Additionally, we created a system of PPIs that was especially targeted at the DEGs. We located sub-networks within this network and highlighted significant hub genes. We developed a network that shows how proteins and drugs interact. Finally, as the final step in our thorough research, we performed molecular docking and MD simulation tests.

### 2.2 Data preprocessing and identification of DEGs

Using the Network Analyst web server tool (https://www.networkanalyst.ca/), the dataset was evaluated for genes displaying differential expression. The focus of this assessment was to locate genes with statistically significant expression variations (Clough and Barrett, 2016). Rows representing specific gene entries and columns representing the various samples made up the dataset’s structure. We used experimental data to determine the inclusion of 10 healthy samples and 12 malignant samples to achieve an equitable distribution. To provide uniformity and clarity throughout the dataset, all gene-probe IDs were also changed into Entrez IDs (Stacklies et al., 2007). We carried out a meta-analysis of the microarray data using NetworkAnalyst, an integrated meta-analysis web application (Xia et al., 2015). Then, we normalized each dataset using a different technique, such as the log2 transformation, quantile normalization, and variance stabilization normalization (VSN). The normality of the data was then confirmed by inspecting principal component analysis (PCA) plots and box plots (Khan et al., 2020). Using a significance threshold of *p* < 0.05, we carried out differential expression analysis on each individual dataset. The Benjamini–Hochberg approach was used to determine an FDR cutoff of ≥2, which we also used. In addition, a *t*-test using the Limma algorithm (LAT) was included in this analysis (Davis, 2007).

### 2.3 Analyzing the functional characteristics of gene sets

The initial ontology analysis of the DEGs was performed using the online bioinformatics program DAVID v6.8 (https://david.ncifcrf.gov/). This was done by tagging them with KEGG pathway information and Gene Ontology (GO) terminology, and annotations with a significance level of *p* < 0.05 were considered (Jiao et al., 2012). Both DEGs and hub genes were included in the modules that we uploaded. Next, we set up several characteristics, including mode-function, species (*Homo sapiens*), molecular function, cellular component, and ontology/pathway–biological process. Data from the Gene Ontology and KEGG databases were used as support for this analysis of functional roles and pathway enrichment (Ashburner et al., 2000; Hulsegge et al., 2009). Utilizing the Benjamini–Hochberg approach, we performed an enrichment analysis utilizing the two-sided hypergeometric test. Using a kappa score of 0.96, the analysis was conducted, and the enrichment was calculated using a threshold value greater than 0.005.

### 2.4 Generating a PPI network

We created a PPI network including each DEG using the STRING database (https://string-db.org/) (Szklarczyk et al., 2015). The database currently comprises 67,592,464 human proteins and 20,052,394,041 documented interactions. The interactions were generated using Cytoscape_v3.10.1 and evaluated using a variety of essential variables. The protein interactions were first imported into Cytoscape version 3.10.1, and they were subsequently evaluated using the integrated tools (Chen Y.-C. et al., 2013). For the combined score to be deemed significant, it must be less than 0.75 (medium confidence score) (Oany et al., 2021). We searched for the direct interactions between each DEG and its first-level interactors. The network design was changed to only include the primary DEGs after a complete dataset with data on both DEGs was imported. Zero-order interactions were specifically chosen to enable exact PPI visualization while removing the crowded and complex network appearance commonly known as the hairball effect (Xia et al., 2014). The network can be analyzed and compared using several different topological variables. Even though Cytoscape is open-source, it has a “NetworkAnalyzer” capability that can be used to examine protein/gene networks. NetworkAnalyzer was used in this study to evaluate significant characteristics such as clustering coefficient, power law-conforming node distribution, node degree distribution, network centralization, and density. These evaluations were carried out to separate the features of the three generated networks (Ma et al., 2011).

### 2.5 Selection of central hub proteins from the PPI network

In order to show and explore the generated PPI networks, Cytoscape version 3.10.1 (https://cytoscape.org/) was used (Shannon et al., 2003). The degree of nodes in the PPI network is determined by the number of edges to which they are connected. Hub genes are identified as nodes with high degree values (Ghafouri-Fard et al., 2023). We mapped the hub genes to examine their PPI details. Essentially, CytoHubba is a well-known integrated Cytoscape tool that analyzes the attributes and ranks the nodes correspondingly (Pan et al., 2023). It uses 11 approaches to examine the functionality of the network, including locating hub genes or network nodes. Therefore, we used CytoHubba to identify hub genes that would make viable novel therapeutic targets for glioblastoma multiforme tumor therapy.

### 2.6 Correlation between transcription factors and regulatory networks

The relationship between transcription variables and their related target genes was established using the X2K eXpression2Kinases (X2K) online application, which can be found at http://www.maayanlab.net/X2K/. This server received as input the whole set of DEGs, along with their individual gene symbols (Chen E. Y. et al., 2013). Established Fisher test *p*-values were used to determine the 10 most notable transcription factors (TFs) and kinases, as well as their enrichment scores. These results came from the TF and kinase module, which was built using information from the ChEA69 database to build the ChIP-X. A regulatory network was created, and Cystoscope was then used to view the “graphml” file (Brandes et al., 2001). It is ensured that the regulatory network has enough interconnected nodes throughout the network development process. If there is a gap in the pathway between kinases and TFs, the system automatically widens it to allow TFs to bind with enough intermediary proteins.

### 2.7 Hub gene survival and expression profile analysis

The Gene Expression Profiling Interactive Analysis (GEPIA2) (http://gepia2.cancer-pku.cn/) (Tang et al., 2017) is an extensive online platform that quickly and adaptably offers a variety of features based on information from The Cancer Genome Atlas (TCGA) and Genotype-Tissue Expression (GTEx). For genes that display differential expression in a particular cancer sample, GEPIA2 evaluates both the effect on survival and the analysis of expression patterns. The overall survival impact of hub genes in GBM was assessed using the GEPIA2 single-gene analysis by calculating the log-rank *p*-value and the 95% confidence interval-based hazard ratio (HR) (Schröder et al., 2011). The hub genes, on the other hand, were chosen according to their relative levels of expression, with a log2FC cutoff value of less than 1 and a *q*-value cutoff of less than 0.01.

### 2.8 Construction of the protein and drug interaction network

The analysis focused on the top 10 hub genes to investigate gene–drug interactions. DrugBank version 5.1.10 (https://go.drugbank.com/), which was integrated with the NetworkAnalyzer program, provided information about the drugs and their corresponding targets. It has 16,222 medication entries in total, of which 2,751 are approved small-molecule pharmaceuticals, 1,604 are approved biologics (including proteins, peptides, vaccines, and allergens), and 134 are approved as nutraceuticals. More than 6,722 investigational medications are also now in the discovery stage. The data entry of each drug is detailed, offering a substantial quantity of information in its 200 or more data fields. This dataset includes information on a number of different topics, such as the chemical makeup and characteristics of the medicine and specifics regarding the target or targets (Wishart et al., 2018).

### 2.9 Molecular docking

Prior to docking, the 2D structures of all possible compounds as inhibitors against the target protein were sketched and minimized using MM2 Force-Field of ChemOffice 2012. Under the Tripos force field (TFF) of UCSF Chimera, a monomer of the target MMP9 protein (pdb id: 5th6) was assigned with Gasteiger charges and minimized for 1,500 steps, which may be divided into 750 of conjugate and 750 of the steepest descent. Molecular docking performed via PyRx 0.9 was used. Molecular docking is an *in silico* approach for determining the binding modes and affinities of two molecules. In the current work, Arg56-N, a charged amino acid, was employed as a binding site residue to dock a molecule with a protein-specific site. A total of four top ligands were docked in the MMP9 protein active site. PyRx 0.9 (Dallakyan and Olson, 2015) software was used to investigate the binding free energies of the docked complexes. For each molecule, 10 solutions were prepared and screened for the best docked pose with high binding affinity. Further investigation was performed to check the binding interactions among the inhibitor moieties and the atomic residues of the protein active site. This was inferred via Discovery Studio (Biovia, 2017), where hydrogen bonds and other active bonds were observed.

### 2.10 MD simulation analysis

First, docked complicated system preparatory simulation was carried out. For protein synthesis, AMBER16 (Case et al., 2016) force fields ff03.rl, GAFF (Sprenger et al., 2015), and ff14SB (Case et al., 2014) were utilized, and the system was solvated within a three-point transferable intermolecular potential (TIP3P) (Brice and Dominy, 2013) water box of 8.0. Minimization was used to remove unfavorable conflicts. For minimization, a conjugate gradient with 1,000 steps and the steepest decent technique were used at a value of 8. Heating was carried out for 10-ps using the Langevin dynamics method for temperature control; at a constant temperature of 300 K, 100-ps equilibration is necessary before the manufacturing run begins. The total energy remains constant during equilibration, whereas kinetic and potential energies change. The docked complex was produced in 200-ns increments, followed by equilibration. Periodic boundary conditions were suggested in the simulation box using a canonical ensemble. To keep the temperature constant, the Berendsen coupling integration approach was applied (Berendsen et al., 1984). The AMBER16 PTRAJ (Process TRAJectory) (Roe and Cheatham, 2013) module was used to generate output files for result analysis. PTRAJ was used to calculate four attributes, and graphical representations were examined in xmgrace (Srivastava et al., 2021).

#### 2.10.1 Binding free energies

The MM(PB/GB)SA approach, which was used in AMBER16, was used to calculate the binding free energy of the top five systems (Case et al., 2016; Srivastava et al., 2021). After extracting 1,000 frames from the simulation trajectories, the AMBER MMPBSA.py module was used for a comprehensive analysis. The MM(PB/GB)SA approach takes into account the difference between the complex free energy and the individual free energies of the ligand and receptor when computing binding free energy. To eliminate false positive results, avoid the limits of one method, and cross-validate the results, binding free energies were estimated using two alternative methods. An implicit solvent system is used by MM(PB/GB)SA to fill the space created by the ligand decoupling process. According to Woods et al. (2004), the water molecules that are inserted into the cavity that is created interact with the protein active site and greatly increase the total binding free energy.

## 3 Results

### 3.1 Assurance of quality and principal component analysis

**FIGURE 2:**
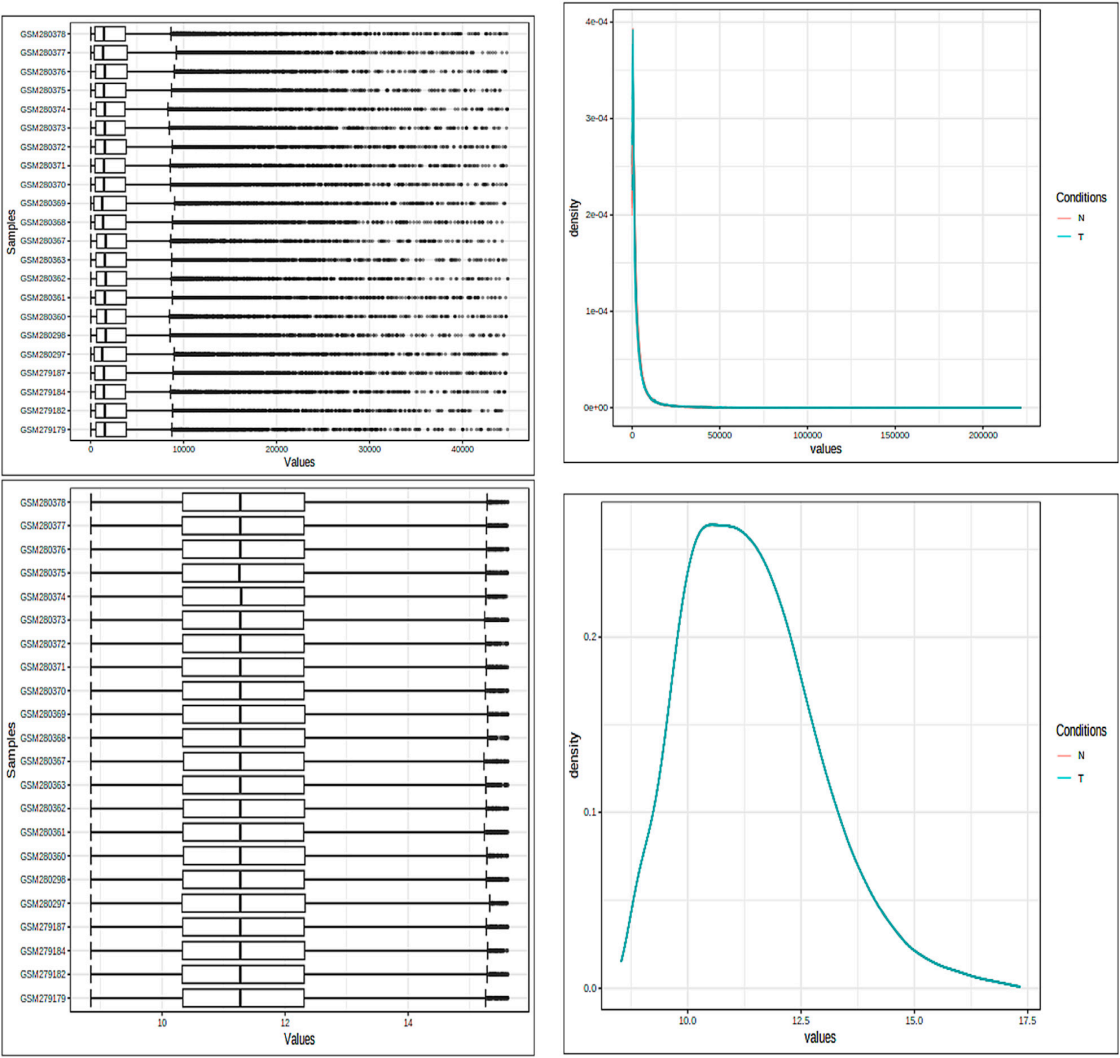
Both box plots and density plots are used to visualize the samples. These graphs are shown in the image both before and after the normalizing process. For mean correction and noise removal, variance stabilization normalization (VSN), followed by quantile normalization, was used.

The fundamental components of a biological system are genes and the products they produce, which interact randomly to form a complex network (Alamri and ul Qamar, 2023). Understanding immunology, the mechanisms of defense, signaling tasks, modes of transportation, and the onset of diseases more thoroughly can be accomplished by analyzing both genes and protein expression. Specifically, in situations where the variation is subject to fluctuations, the VSN, followed by the quantile normalization method, was used to standardize the expression data from microarray analysis. Box plots, density plots, and the data means are shown in Figure 2 both before and after the normalizing process. The figure illustrates how the normalization procedure produces constant means across all samples and successfully removes any noise that may have been present in the data. The PCA of the data revealed clear clusters. PCA was used to separate the controlled and mutant samples of the dataset into discrete clusters based on the levels of gene expression.

### 3.2 Investigation of DEGs and network analysis of PPIs

**FIGURE 3:**
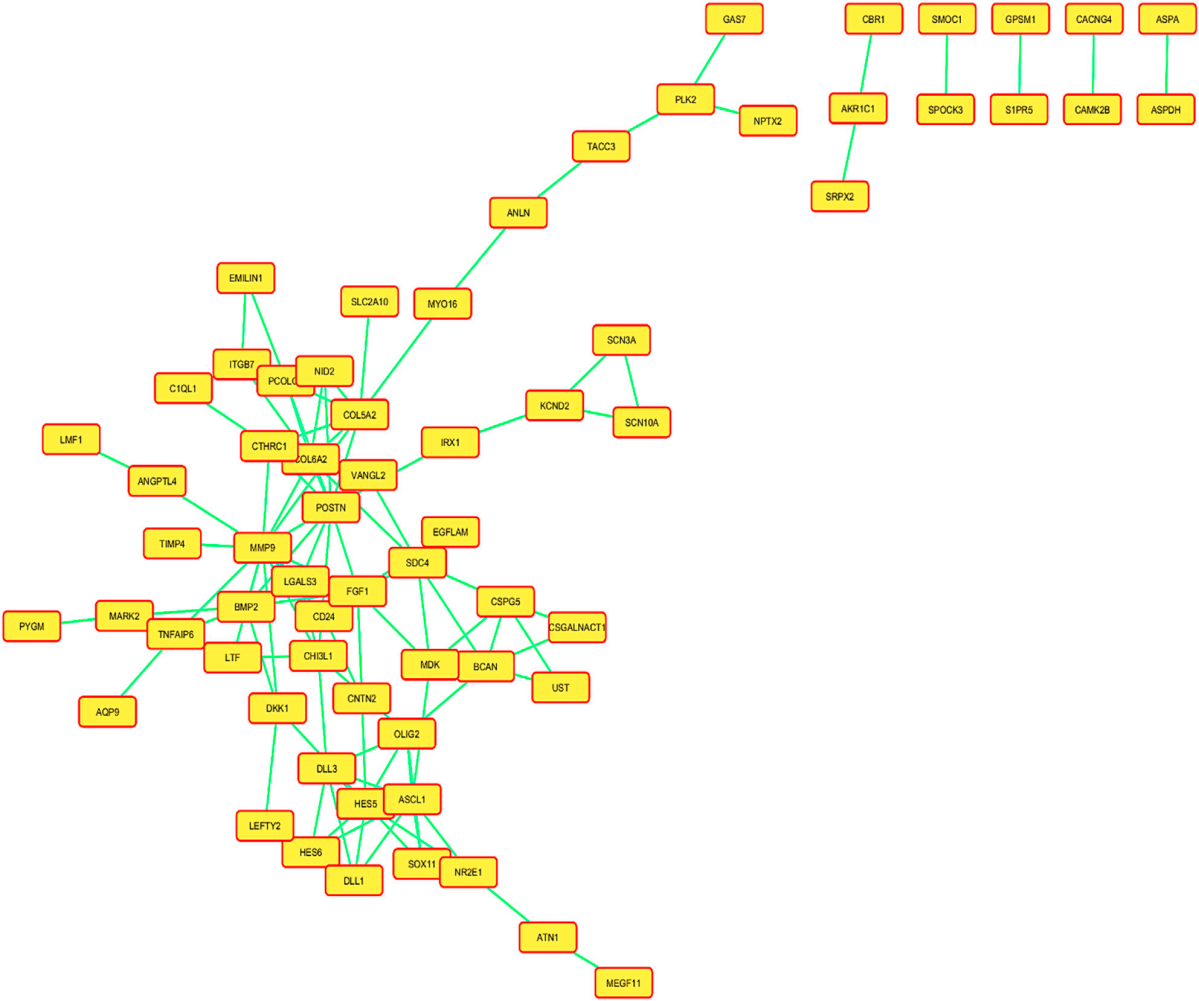
Network of the protein–protein interactions (PPIs) of all the mapped differentially expressed genes (DEGs), and a confidence level of 0.400 was used to build this network using information from the STRING database.

Several statistical tests were run on the datasets for both the control and disease state, including Student’s t-test, Benjamini–Hochberg technique, and Pearson’s correlation test. A total of 132 DEGs were discovered Utilising these investigations (Table 1). A total of 13 of these DEGs showed upregulation, while only 29 showed downregulation individually, and the rest of genes were insignificant. Using the adjusted *p*-value, the final ranking of the DEGs was established (Table 2). The network of PPIs is very important for understanding the workings of cellular networking. To assess how serious the issue is, changes in the protein cellular network in both healthy and diseased states are useful. In the present investigation, proteins and genes are represented by nodes, while interactions between these proteins and genes are represented by edges. We mapped these genes and created an interaction network for the identified DEGs. The mapped DEGs were shown graphically using Cytoscape. All interactions involving the DEGs were retrieved from the STRING database using a moderate confidence level of 0.40. The network created utilizing every DEG found is shown in Figure 3.

**TABLE 1:** Top 10 DEGs in the protein–protein interaction network from the STRING database ranked by the degree method.

Rank	Gene name	Score	logFC	Adjusted *p*-value
1	Matrix Metallopeptidase 9 (*MMP9*)	14	1.4035	0.21842
2	Periostin (*POSTN*)	11	1.5999	0.25854
3	Hes Family BHLH Transcription Factor 5 (*HES5*)	9	−1.0343	0.18261
4	*Achaete-Scute Family BHLH Transcription Factor 1 (ASCL1*)	8	−1.0575	0.18658
4	*Collagen Type V Alpha 2 Chain (COL5A2*)	8	1.2027	0.21842
6	*Fibroblast Growth Factor 1 (FGF1*)	7	1.0553	0.17267
6	*Collagen Type VI Alpha 2 Chain (COL6A2*)	7	1.3605	0.20412
8	*Syndecan 4 (SDC4*)	6	1.2696	0.073206
8	*Bone Morphogenetic Protein 2 (BMP2*)	6	−1.0586	0.11314
8	*Delta Like Canonical Notch Ligand 3 (DLL3*)	6	−2.0239	0.028407

**TABLE 2:** Identification of proposed drugs against the hub proteins retrieved from the database.

S. no.	Protein	UniProt id	Drug name	DrugBank id	Groups
	*Matrix Metallopeptidase 9 (MMP9*)	P00390	Carmustine	DB00262	Approved
		Q9H169	Lomustine	DB01206	Approved
		P14780	Marimastat	DB00786	Investigational
		A0A6M3QJP3	Temozolomide	DB00853	Approved
	*Periostin (POSTN*)	P46721	Prednisolone	DB00860	Approved
		P35354	Salicylic acid	DB00936	Approved
	*Hes Family BHLH Transcription Factor 5 (HES5*)	……………	……………	……………	……………
	*Achaete-Scute Family BHLH Transcription Factor 1 (ASCL1*)	……………	……………	……………	……………
	*Collagen Type V Alpha 2 Chain (COL5A2*)	H6U5S3	Clofarabine	DB00631	Approved
	*Fibroblast Growth Factor 1 (FGF1*)	P05067	Flutemetamol F-18	DB09151	Approved
	*Collagen Type VI Alpha 2 Chain (COL6A2*)	……………	……………	……………	……………
	*Syndecan 4 (SDC4*)	……………	……………	……………	……………
	*Bone Morphogenetic Protein 2 (BMP2*)	……………	……………	……………	……………
	*Delta Like Canonical Notch Ligand 3 (DLL3*)	……………	……………	……………	……………

**FIGURE 4:**
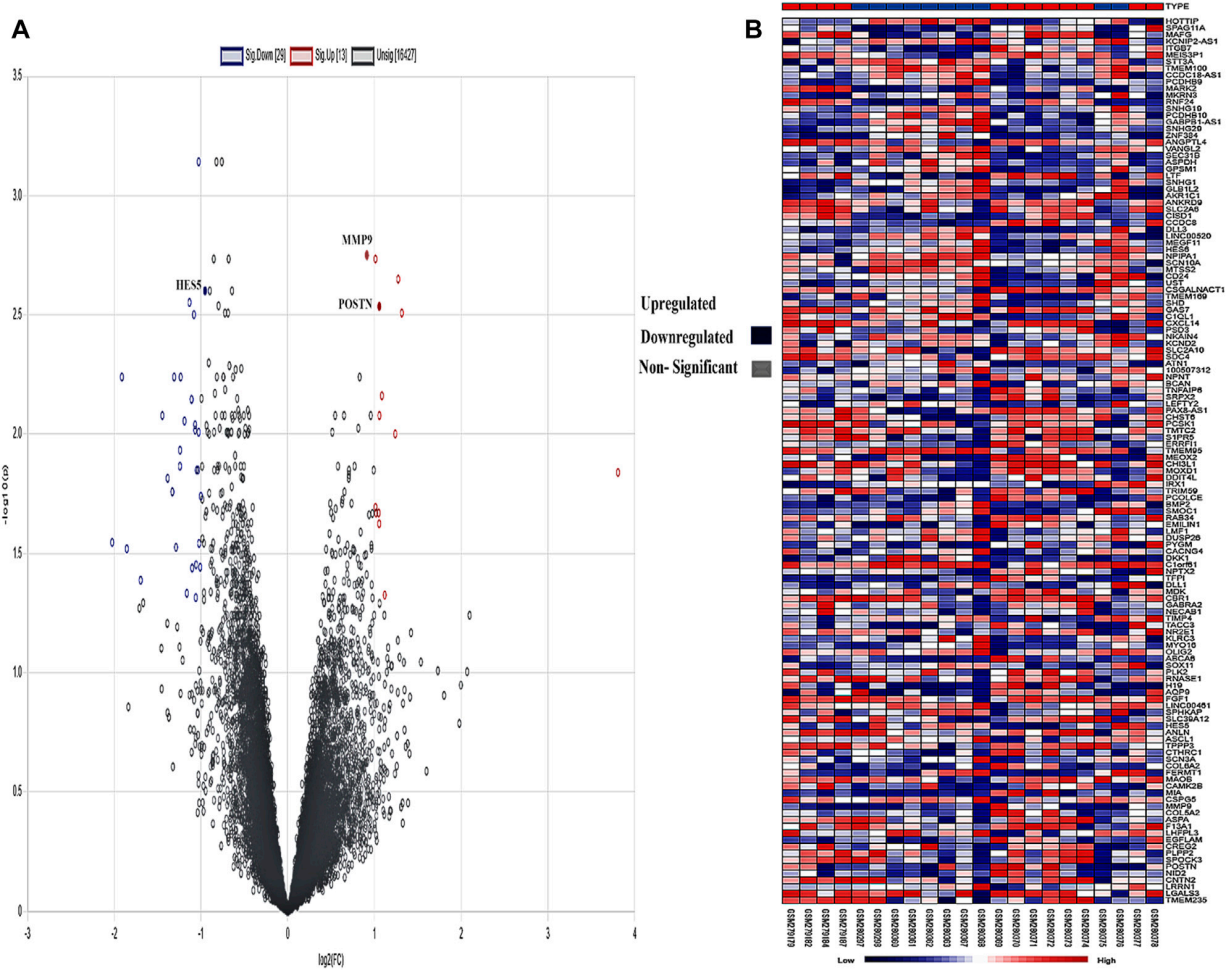
**(A)** Intensity distribution of each gene is shown on the volcano plot. Red indicates genes that are elevated based on a false discovery rate (FDR) threshold greater than 2, blue indicates genes that are downregulated, and gray indicates non-significant genes in the samples. **(B)** The genes are shown against each sample in the form of a heatmap, and the legend shows the distribution of expression values, which range from low (blue) to high (red).

A created network consisted of 66 nodes linked by 105 edges. The network topological metrics showed that there were significant interactions within it, with an average node degree of 1.75 and an average local clustering coefficient of 0.304. Both a volcano plot and a heatmap are shown in Figure 4 to represent the DEGs.

### 3.3 Analysis of the KEGG pathway and GO function enrichment

The top 10 results of the enrichment analysis for DEGs that are upregulated and downregulated in the context of GO analysis were determined using the DAVID database, including topics such as cellular components, molecular functions, biological processes, and KEGG pathways, as described by GO. A highly significant criterion for the terms used in the analysis was the FDR. Our analysis of the GO cellular components (CCs) of the DEGs revealed a highly significant and distinctive distribution pattern for our proteins, including extracellular space, extracellular region, extracellular matrix, collagen trimer, cell surface, basement membrane, voltage-gated calcium channel complex, microtubule bundle, synapse, tertiary granule lumen, perinuclear region of the cytoplasm, GABAergic synapse, glutamatergic synapse, sarcoplasm, and voltage-gated sodium channel complex. We found 15 key functional categories for which our proteins actively participate in terms of molecular functioning (MF), such as extracellular matrix structural constituent, growth factor activity, heparin binding, carbohydrate binding, collagen binding, calcium ion binding, sequence-specific double-stranded DNA binding, identical protein binding, dehydroascorbic acid transporter activity, oxidoreductase activity, integrin binding, D-glucose transmembrane transporter activity, co-receptor binding, metalloendopeptidase inhibitor activity, and protein dimerization activity.

Furthermore, the significant KEGG pathways are primarily connected to cancer-related processes, and the reported pathways, which include ECM–receptor interaction, Notch signaling pathway, breast cancer, and endocrine resistance, are highly enriched.

The enhanced biological processes (BPs) identified in DEGs include extracellular matrix organization, cell adhesion, negative regulation of neuron differentiation, Notch signaling pathway, positive regulation of osteoblast differentiation, skeletal system development, negative regulation of cardiac muscle cell differentiation, regulation of neurogenesis, positive regulation of osteoblast proliferation, central nervous system myelination, cell–matrix adhesion, astrocyte differentiation, cell migration, inner-ear receptor stereocilium organization, positive regulation of neural precursor cell proliferation, oligodendrocyte development, positive regulation of oligodendrocyte differentiation, positive regulation of sprouting angiogenesis, nervous system development, positive regulation of angiogenesis, positive regulation of the transforming growth factor beta receptor signaling pathway, and negative regulation of the canonical Wnt signaling pathway.

### 3.4 Hub gene identification

**FIGURE 5:**
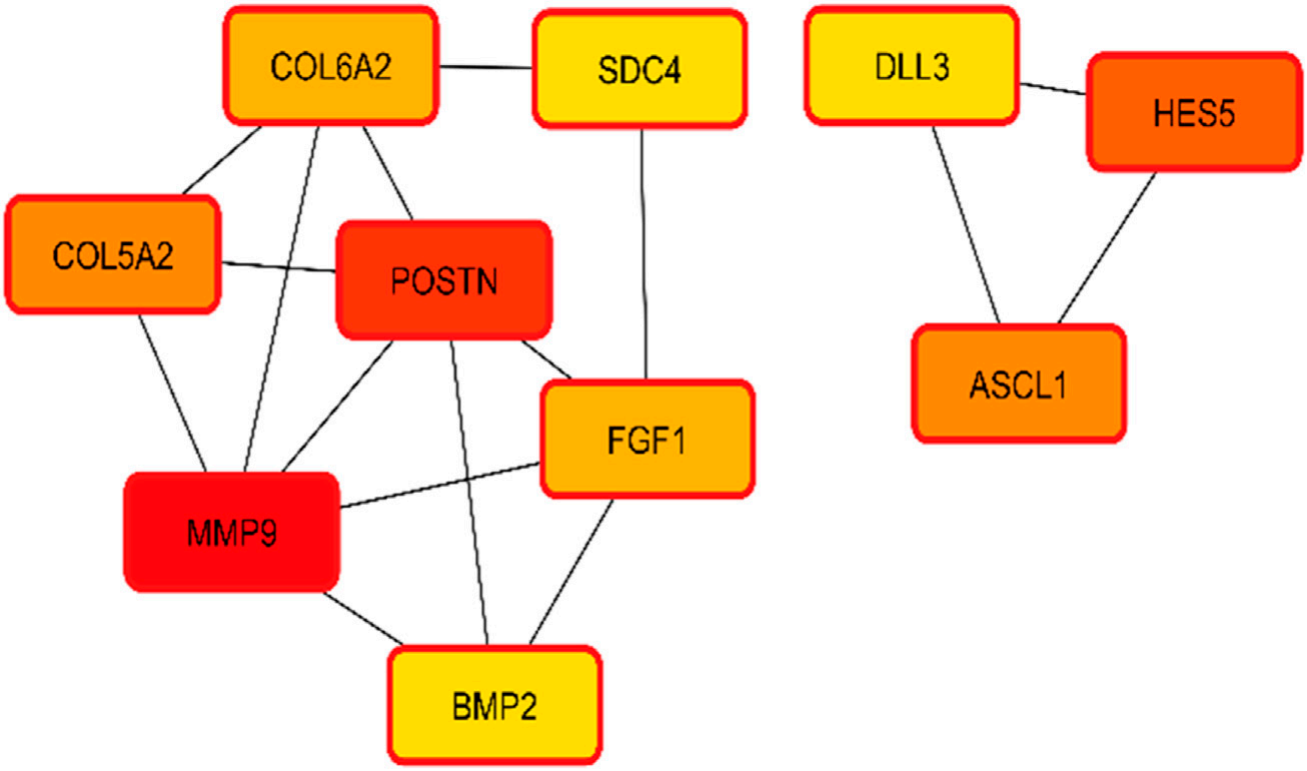
Hub gene identification and interactions based on the degree of color and score. Higher scores having strong interaction with other targets are shown in red, followed by orange and yellow.

The interactions of hub genes with the red nodes determined and ranked the nodes according to their degree of significant connection. Nodes having a degree value > 10 were classified as hub nodes using CytoHubba, which calculated the node degrees (Table 1). *MMP9* had the highest degree, followed by 11 *POSTN* and Hes family BHLH transcription factor 5 (*HES5*) with a 9-degree score. Herein, achaete-scute family BHLH transcription factor 1 (*ASCL1*)*,* collagen type-V alpha 2 (*COL5A2*), fibroblast growth factor 1 (*FGF1*), collagen type-VI alpha 2 (*COL6A2*), syndecan 4 (*SDC4*), bone morphogenetic protein 2 (*BMP2*), and delta-like canonical notch ligand 3 (*DLL3*) were among the additional genes revealed to have lower degree values. The shortest path network of these hub gene interactions showing strong interactions based on the degree of score and color is given in Figure 5.

### 3.5 Transcription factor analysis

**FIGURE 6:**
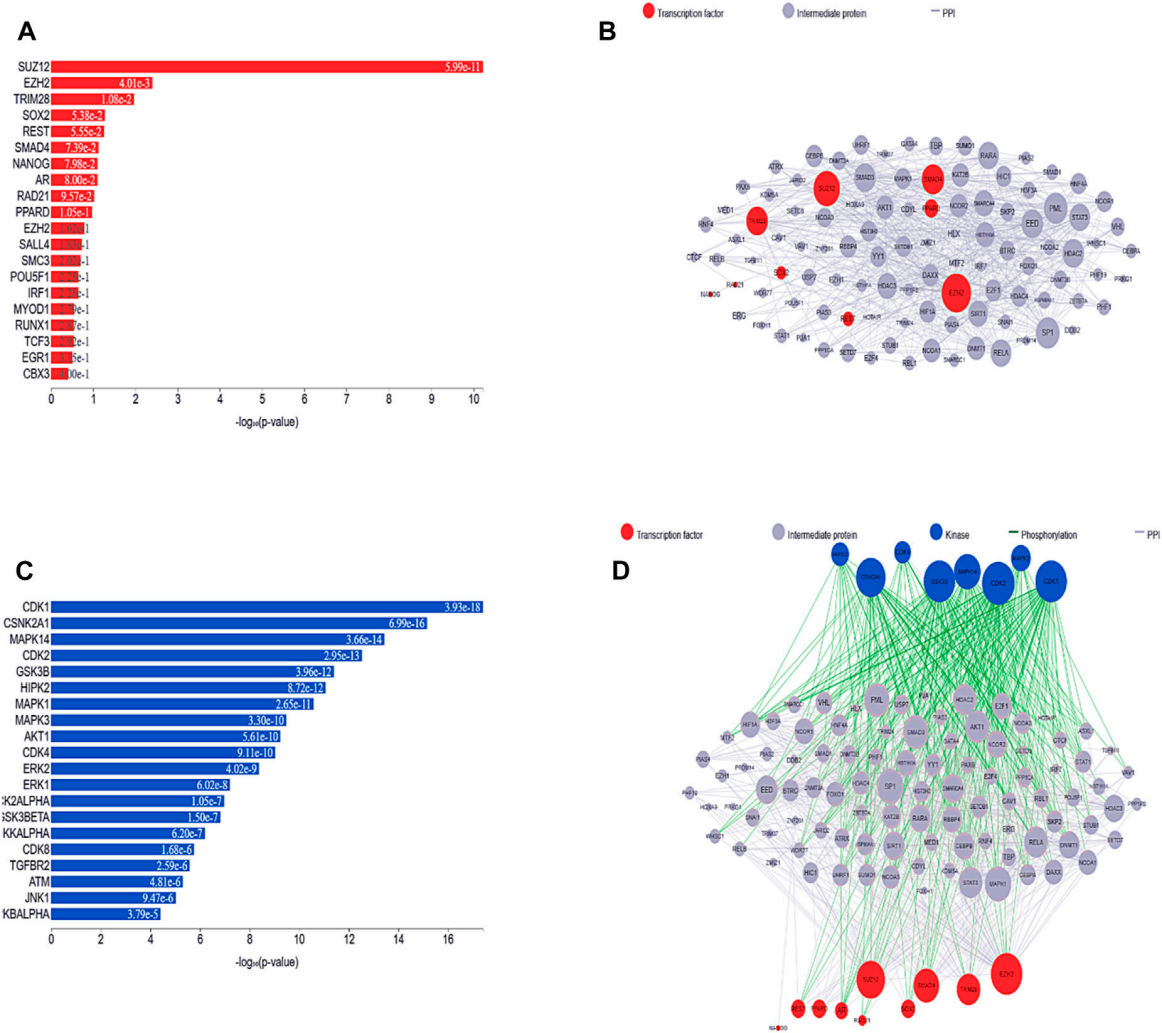
**(A)** Predicted transcription factors identified in the list of DEGs are shown in a bar chart. The height of each bar, which represents a transcription factor, reflects that factor score as determined by the hypergeometric *p*-value. **(B)** A subnetwork illustration is displayed, emphasizing interrelated transcription factors and the interacting proteins that go along with them. Pink nodes represent transcription factors, while gray nodes represent the proteins that connect them. The degree of connectedness between the nodes in this network determines how big they are. **(C)** Bar graph ranking the top predicted kinases. Hypergeometric *p*-values are used in the graph to display the scores of these kinases. **(D)** By investigating known protein-protein interactions between mutually overlapping differentially expressed genes (DEGs), one can gain insight into the involvement of transcription factors and kinases in the upstream pathway.

The most well-known TFs and protein kinases connected to DEGs were identified in this analysis based on their contributions to the development of regulatory network activities. TFs, kinases, and transient proteins involved in their development were connected to create a network that served as a regulatory complex. First, we used ChIP-seq experiments (ChEA) to identify integrated target genes for transcription factors, which were then used to predict the most important TFs, which were then mapped onto PPI networks. Figures 6A,B depict the TFs predicted here and the PPI network. Based on the hypergeometric *p*-value, the main transcription factors in this case are SUZ12 polycomb repressive complex 2 subunit (*SUZ12*)*,* enhancer of zeste 2 polycomb repressive complex 2 subunit (*EZH2*), (tripartite motif containing 28 (*TRIM28*), SRY-box transcription factor 2 (*SOX2*), (RE1 silencing transcription factor (*REST*), and (SMAD family member 4 (*SMAD4*). We also identified and subsequently added into the PPI network kinases that are most likely to behave as regulators for the extended PPI network, as shown in Figures 6C,D. The major kinases in these DEGs identified based on the hypergeometric *p*-value were cyclin-dependent kinase 1 (*CDK1*), casein kinase 2 alpha 1 (*CSNK2A1*), mitogen-activated protein kinase 14 (*MAPK14*), cyclin-dependent kinase 2 (*CDK2*), glycogen synthase kinase 3 beta (*GSK3B*), homeodomain interacting protein kinase 2 (*HIPK2*), mitogen-activated protein kinase 1 (*MAPK1*), mitogen-activated protein kinase 3 (*MAPK3*), AKT serine/threonine kinase 1 (*AKT1*)*,* cyclin-dependent kinase 4 (*CDK4*), cyclin-dependent kinase 2 (*ERK2*), mitogen-activated protein kinase 3 (*ERK1*), casein kinase 2 alpha 1 (*CK2ALPHA*), glycogen synthase kinase 3 beta (*GSK3BETA*), and component of inhibitor of nuclear factor kappa B kinase complex (*IKKALPHA*). All the TFs and kinases identified with their scores are given in Supplementary Table S1.

### 3.6 Identified hub gene survival and expression analysis

**FIGURE 7:**
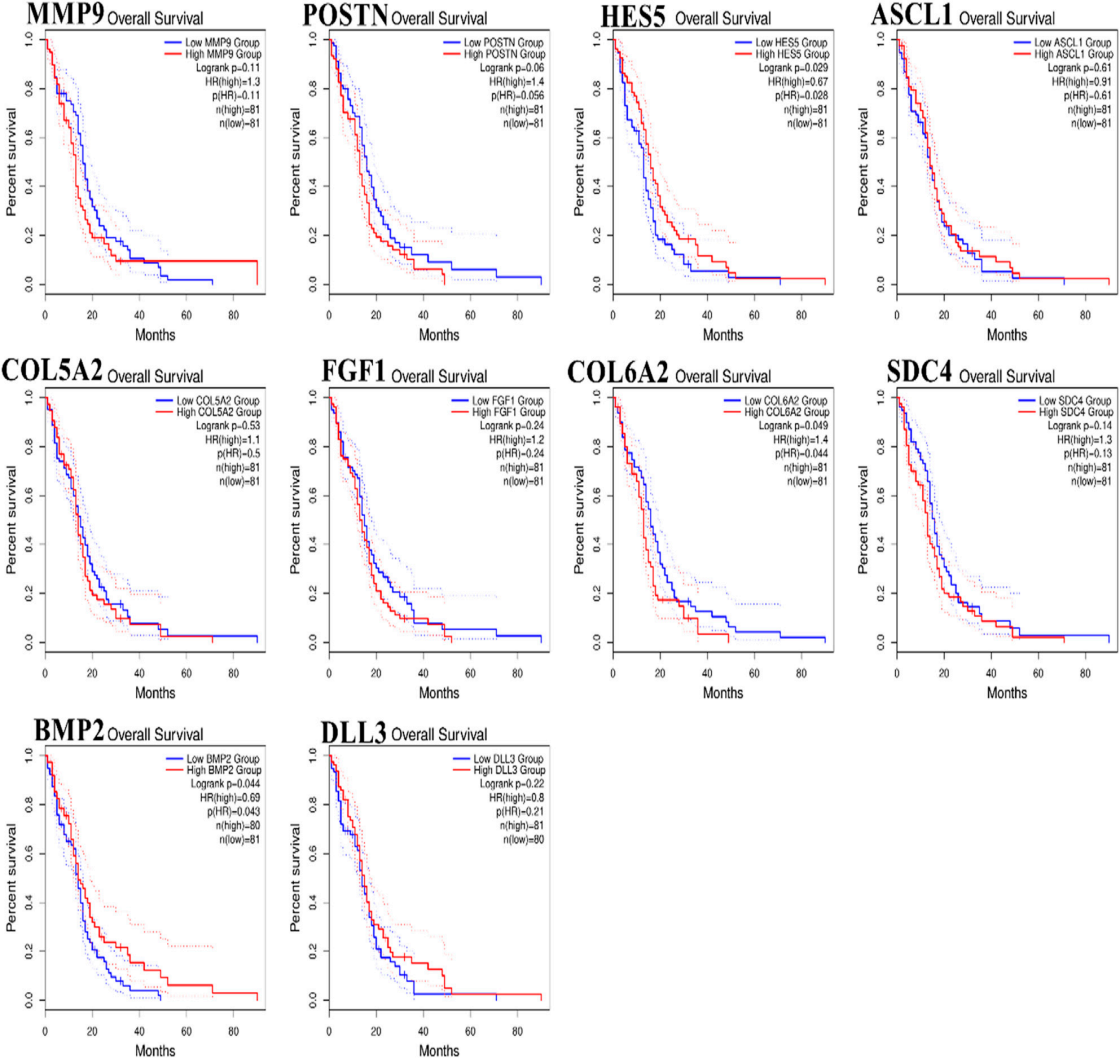
Hub genes expressed in glioblastoma multiforme (GBM) patients were subject to Kaplan–Meier overall survival analysis. Using The Cancer Genome Atlas database as a base, curves were produced using Gene Expression Profiling Interactive Analysis (*p* ≤ 0.01).

**FIGURE 8:**
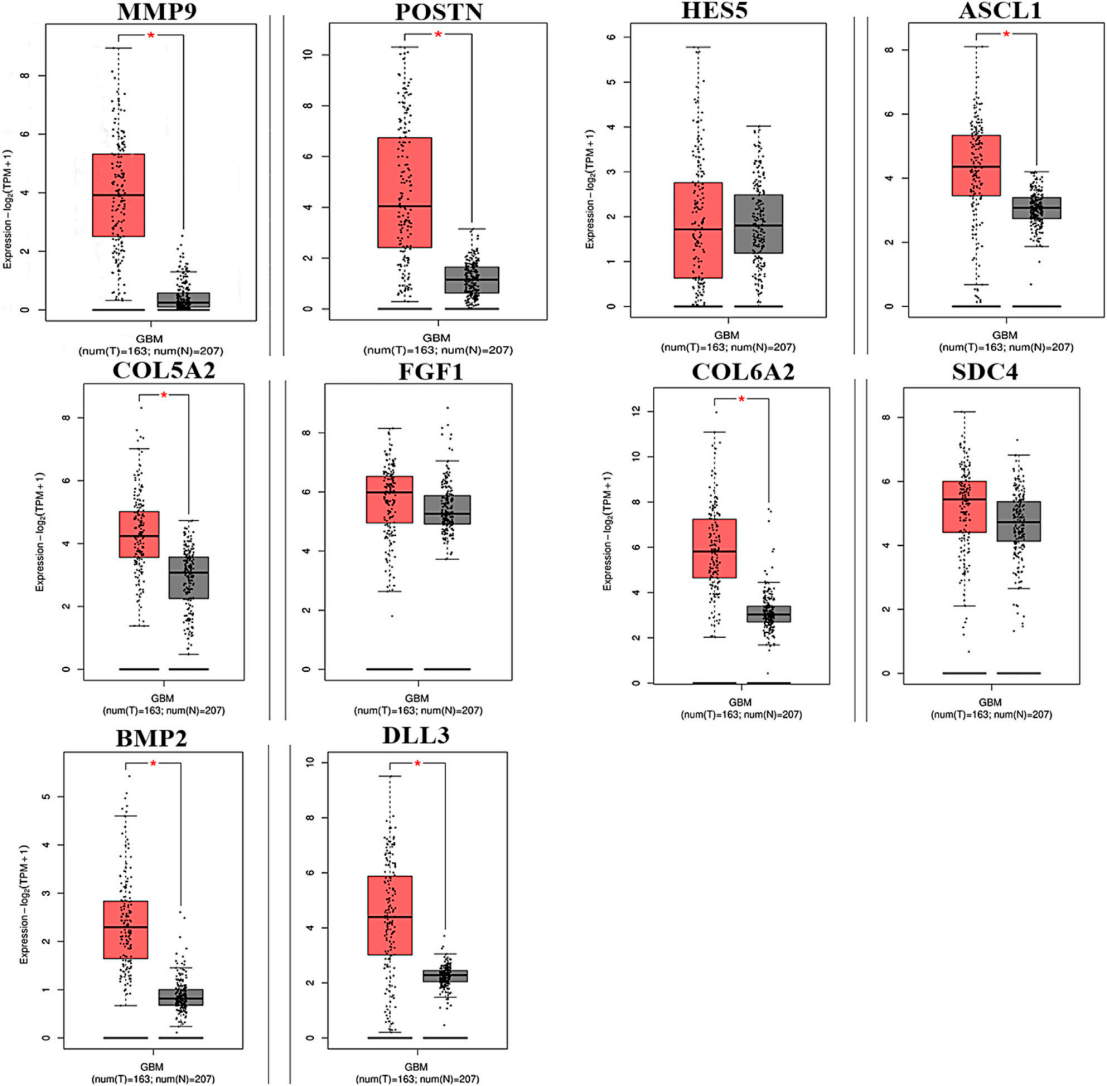
Hub genes expressed in GBM at different levels of relative expression. Relative gene expression levels for the tumor and normal samples are shown in red and black boxes, respectively. A total of 163 tumor samples and 207 normal samples from GEPIA are represented on the *y*-axis as the relative expression levels of genes in terms of log2 (TPM +1) (*p* ≤ 0.01) transcribing units per million (TPM).

To analyze the overall impact on the survival of 10 important hub genes chosen from both the upregulated and downregulated DEGs, *GEPIA* survival evaluation was used. Figure 7 shows the findings of the current study. COL6A2 had a hazard ratio (HR) of 1.4 and was the only one of the 10 genes under investigation to show decreased overall survival in the group with increased expression. On the other hand, from the expression level of the hub genes, we identified that the nine hub genes *MMP9*, periostin (*POSTN*), *ASCL1*, *COL5A2*, *FGF1*, *COL6A2*, *SDC4*, *BMP2*, and *DLL3* were downregulated in the normal cells, and only one hub gene, *HES5*, was upregulated in the tumor cells (Figure 8).

### 3.7 Protein–drug interactions

Following the identification of the top hub genes, the DrugBank database was searched for possible therapeutic candidates. In this study, we investigated each target and, using manual searches, identified the drugs related to them. The search panel included medication from a range of categories, including FDA-approved, investigational, and nutraceuticals. We manually searched for medications that interacted with each hub gene-recognized protein. In total, eight medications were found. Four drugs for MMP9, two for POSTN, and one each for COL5A2 and FGF1 were found, while no potential drugs were found for the rest of the targets. The identified drugs for all these targets are given in Table 2.

### 3.8 Molecular docking

**FIGURE 9:**
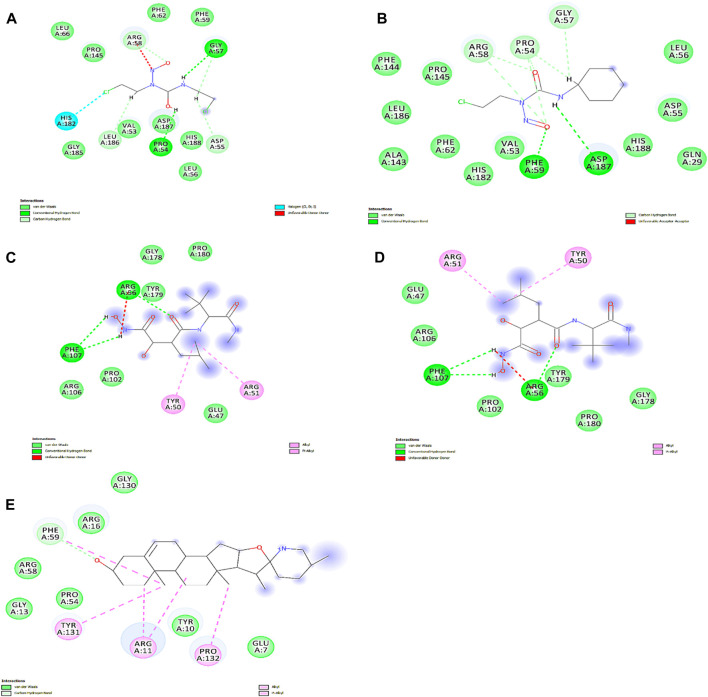
Interactions of docked complexes. **(A)** Carmustine complex, **(B)** lomustine complex, **(C)** marimastat complex, and **(D)** temozolomide complex. **(E)** Control solasodine with the target protein.

We obtained the four best compounds from the database against the target hub gene. All the compounds were docked into the target protein active site, with the coordinates of the Arg56: N atom set as the center of binding. A radius of 10 Å was set around Arg56: N, and 10 iterations were generated for each docked compound. Docked scores were used to assess chemical binding affinity for protein activity. The docked score for the complexes carmustine, lomustine, marimastat, temozolomide, and solasodine (control drug) with the MMP9 protein is −6.3, −7.4, −7.7, −8.7, and −5.7 kcal/mol, respectively. Furthermore, major residues of the protein active site implicated in hydrogen bonding, such as Glu47, Arg56, Tyr179, Pro102, Phe107, Arg106, Pro180, Asp187, Phe59, Gl57, Pro54, and Val53, were involved in binding forces, as shown in Figure 9.

Some of the residues indicated possible hydrophobic interactions. The theoretical activity of a compound requires both hydrogen and hydrophobic contacts. Furthermore, numerous additional interactions were observed, which must be considered for the computational efficacy of the compound. Thus, it has been inferred that these chemical moieties are oriented in such a way that they bind along the channel of the active pocket. Thus, it prevents the total substrate access to the active site during the metabolic pathways and inhibits the final production of cancerous substances.

### 3.9 MD simulation

**FIGURE 10:**
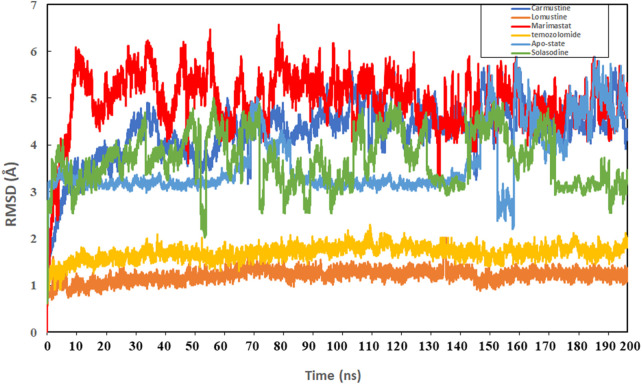
Graphical representation of the root mean-square deviation (RMSD) graph of the four simulated systems with a time interval of 200 ns

**FIGURE 11:**
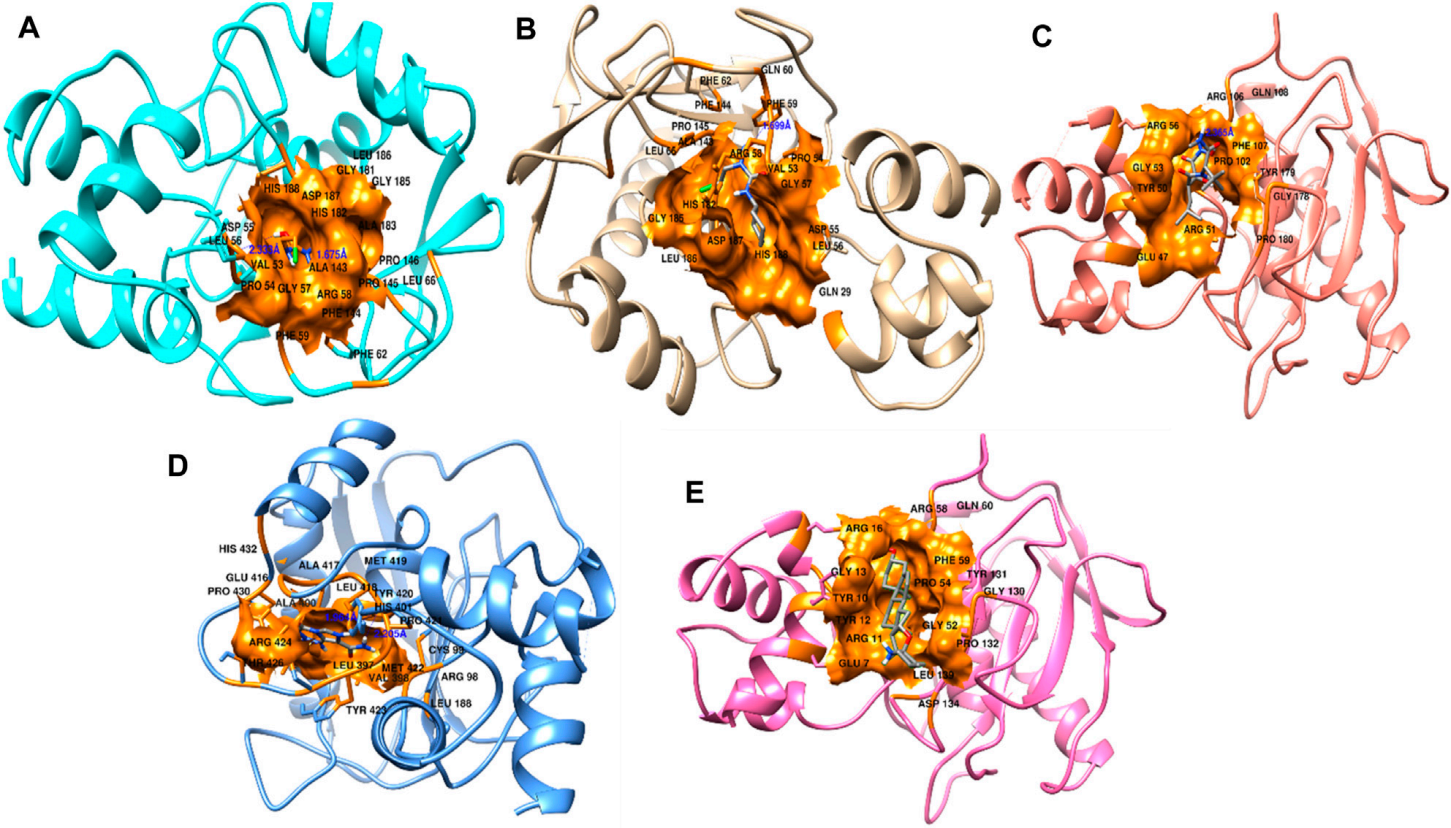
Root-mean-square fluctuation (RMSF) values of complexes in the Apo state and in the control system.

**FIGURE 12:**
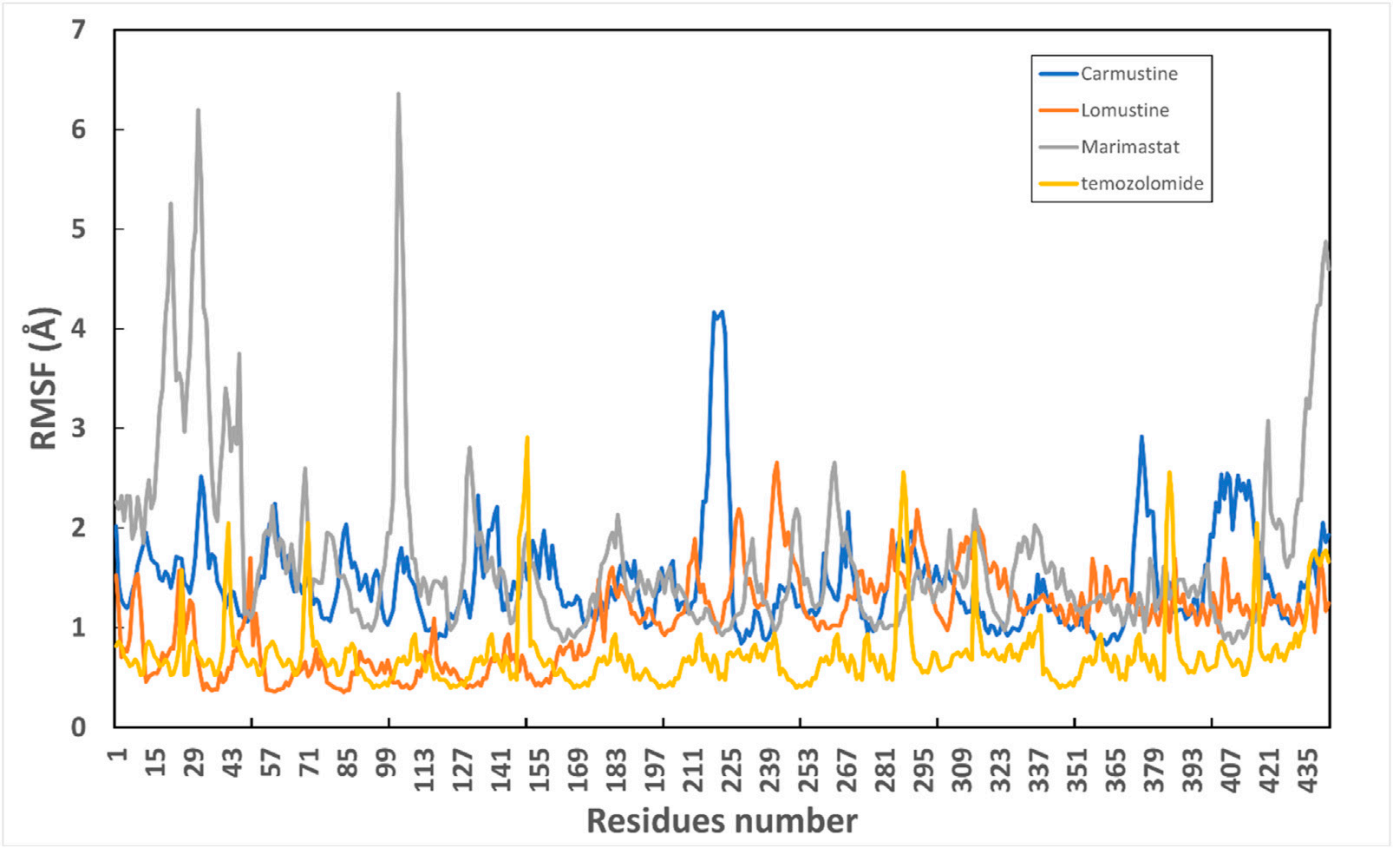
Binding pose of the interacting linkages at the end of simulation time intervals. Blue depicts hydrogen bonds inside the pocket residues along with other active residues throughout the simulation time intervals.

**FIGURE 13:**
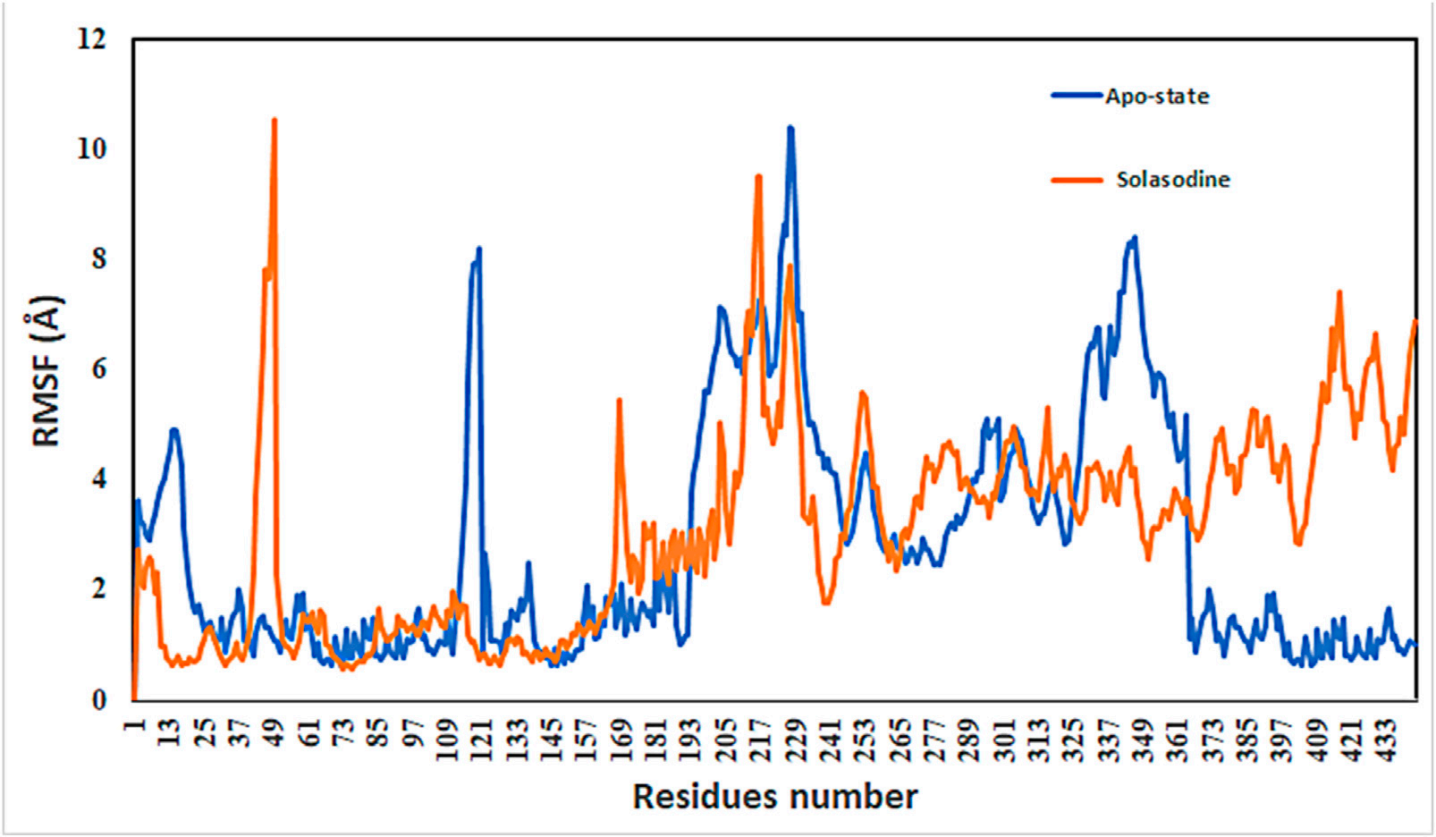
Graphical representation of RMSF of all four complex systems with the number of residues throughout simulation time intervals.

Protein conformational dynamics are the most essential aspect linked with their function. The functional information of a protein molecule is encoded in its structure. To comprehend the functional variability, an understanding of the structure is required (Karplus and Kuriyan, 2005). The current work used MD simulation to investigate the conformational aspect of protein–ligand interactions and assess the stability of the inhibitor complex system in a real-time environment. Data reduction analyses such as root-mean-square deviation (RMSD) and root-mean-square fluctuation (RMSF) were used to assess the conformational changes and stability index of secondary structure components of the simulated complexes. RMSD describes the backbone analysis Cα atom dynamics of the docked protein during a time scale of 100 ns, and fluctuation was observed at different time intervals among carmustine complex and marimastat complex systems, but a persisting graph of simulation stability was detected among the marimastat and temozolomide complex system. Figure 10 shows that the average RMSD value for the carmustine complex and marimastat complex system is 4.1 Å and 5.03 Å, respectively, whereas the average RMSD for the lomustine and temozolomide complex systems is 1.2 Å and 1.6 Å, respectively. To check the stability of the two carmustine complex and marimastat complex systems, simulation time intervals were extended for a further 100 ns. It was found that the system gains stability after 100 ns and remains equilibrated till 200-ns time intervals. Herein, the average RMSD recorded for both systems was 4.2 Å and 4.9 Å, respectively. It is possible that the natural flexibility of the N-terminal area is the cause of the oscillations shown in the simulation intervals. An overall structure may fluctuate because of the conformational changes introduced by the loop sections inside the N-terminal segment. These variations could be caused by the dynamic interactions the N-terminal residues with the surrounding solvent or other protein components. Furthermore, the RMSD for the protein target in Apo and complex states with the control inhibitor solasodine results in an average mean square value of 3.61 Å and 3.68 Å, respectively. Fluctuation was recorded in both Apo and complex states with a time scale of 200 ns. This overall fluctuation was then observed in the residual system of the RMSF, where the N-terminal region of the protein target showed high conformational changes. The average RMSF recorded for the Apo state was 2.8 Å. Meanwhile, for the control complex, it was measured as 3.0 Å with a time scale of 200 ns, as shown in Figure 11. The binding poses during simulation time intervals were retrieved for all the complexes at the end of the simulation, where it was inferred that strong hydrogen bond linkages were observed with a time scale of 200 ns, as shown in Figure 12. Overall, the RMSD graph pattern supports any large domain alterations within the protein–ligand complex structural framework of carmustine complex and marimastat complex systems. Herein, the inhibitors remained inside the active site during the initial time frame but moved as the time scale extended till the end of the simulation. On other hand, the ligands lomustine and temozolomide in the complex system were properly supplemented inside the binding site and did not destabilize the protein, as shown in Figure 10. Figure 13 shows RMSF values that detect the structure flexibility and volatility of Cα residues over time. A high pitch of fluctuation was recorded among the carmustine complex and marimastat complex systems. No such fluctuation was observed till 200-ns simulated time intervals, with a minor peak due to the loop region of the protein among lomustine and temozolomide in the complex system. Strong hydrogen bonds were monitored in the active zone of the MMP9 protein target. These residues include Asp187, Phe59, Gl57, and Pro54. The average RMSF of the carmustine complex and marimastat estimated from 200 ns was 1.4 Å and 1.7 Å, with a maximum peak of 4.1 Å and 6.3 Å, respectively, while substantial variations were noted at several residual points, and finally, at the conclusion of the graph for the other stable complex systems, a minor residual point was observed with an average RMSF of 1.0 Å and 0.7 Å, with no such displacement of atomic residues among the helix and sheet regions, which makes them highly stable systems. The binding pocket residues involved in RMSF include His188, Pro54, Asp187, Val53, Leu186, Gly185, Phe59, Phe62, Leu66, Ala143, Arg56, Arg51, Tyr50, Pro102, and Arg106. These residues may change as a result of ligands attaching to the active site. This implies that the flexibility or dynamics of these regions is higher. Active site residues may be involved in greater RMSF for a number of reasons; some of these residues have flexibility, whereas some are compact due to their carbon alpha background.

#### 3.9.1 Binding energy calculation

The binding free energies of all four ligands demonstrate their affinity for the protein active site. They are considered useful assessment criteria for leading chemical discovery and offer mechanistic insights into the binding ligands (Slynko et al., Citation 2016). Strong interactions between enzyme and inhibitor complexes were shown by the MM(PB/GB)SA technique of AMBER16, as shown in Table 3. The entropy computations were discarded because of a convergence issue. It was determined that in the instance of MM(PB/GB)SA, complex formation resulted in a highly favorable electrostatic contribution of −12.48 kcal/mol for carmustine, −16.14 kcal/mol against lomustine, −10.97 kcal/mol for marimastat, −17.29 for temozolomide, and −13.35 kcal/mol for solasodine. Likewise, the dominating electrostatic energy contribution derived from MMPBSA was also identified. Furthermore, a highly beneficial contribution to inhibitor complexes comes from the van der Waal energy values of −25.10 kcal/mol (carmustine), −57.63 kcal/mol (lomustine), −30.98 kcal/mol (marimastat), and −51.17 kcal/mol (temozolomide), and the solasodine complex shows −34.54 from MMGBSA. Similarly, from MMPBSA, the following complexes had favorable van der Waal energies: the solvation energy with a binding energy of 10.63 kcal/mol (carmustine), 9.03 kcal/mol (lomustine), 15.85 kcal/mol (marimastat), and 10.11 kcal/mol (temozolomide), whereas the control solasodine showed 11.23 kcal/mol after 200-ns time intervals. Finally, the total average binding energies regarding MMGB/PBSA were –24.95 kcal/mol for carmustine, –39.74 kcal/mol for lomustine, –31.07 kcal/mol for marimastat, –41.35 for temozolomide, and −28.55 kcal/mol for the control complex. These binding energies show that the simulated complexes lomustine and temozolomide had high binding affinities and remain stable during the whole time scale, which results in building a high ground for the experimentalist.

**TABLE 3:** Binding free energies of all the compounds after 200-ns simulation time intervals.

Parameter	Carmustine	Lomustine	Marimastat	Temozolomide	Solasodine
MMGBSA					
van der Waals energy	−25.10	−57.63	−30.98	−51.17	−34.54
Electrostatic energy	−12.48	−16.14	−10.97	−17.29	−13.35
Total gas phase energy	−32.58	−48.77	−35.95	−55.46	−28.46
Total solvation energy	10.63	9.03	15.85	10.11	11.23
Net energy	−24.95	−39.74	−31.07	−41.35	−28.55
MMPBSA					
van der Waals energy	−25.10	−57.63	−30.98	−51.17	−34.54
Electrostatic energy	−12.48	−16.14	−10.97	−17.29	−13.35
Total gas phase energy	−32.58	−48.77	−35.95	−55.46	−28.46
Total solvation energy	10.17	9.30	15.97	10.37	10.21
Net energy	−24.41	−39.47	−31.98	−41.09	−29.53

## 4 Discussion

Different digital methods are available for analyzing various kinds of genomic data, including the analysis of SNPs, genome-wide association studies (GWASs), and the analysis of gene expression using microarrays. These techniques are essential for gathering key knowledge about diseases, from diagnosis to treatment options (Akcay et al., 2018). All these methods, including genomics, proteomics, transcriptomics, metagenomics, epigenetics, and metabolomics, can be used to collect a variety of data from various phases. They frequently play a crucial role in the development and prediction of biomarkers for prediction and progress evaluation (Khan et al., 2020). PPI network analysis is frequently used to identify prospective therapeutic targets, understand metabolic processes, and shed light on the mechanisms behind various diseases (Hu et al., 2019). The structural relationships between numerous proteins, which might differ between healthy and disease phenotypes, have a significant impact on various biological processes (Ni et al., 2009; Liu et al., 2018). Using microarray gene expression data analysis, one can identify targets for the creation of novel medications by identifying genes that are differentially expressed in the setting of a disease as opposed to the normal condition. The precision and robustness of detecting disease-linked biomarkers are improved by molecular network interactions (Ma et al., 2016). Previous studies have demonstrated the value of such investigations in foretelling the core nodes and their key roles in a number of diseases (Nayarisseri et al., 2013; Khan et al., 2018).

Compared to cases of GBM in advanced stages, the prognosis for early-stage cases is notably better. Through therapies including surgery, chemotherapy, radiation therapy, or a combination of therapies, early-stage patients frequently experience significant cure rates. Patients with advanced-stage glioblastoma multiforme, however, experience extra challenges, mostly as a result of the tough nature of the disease and frequent inability to be cured (Henson, 2006).

In this study, we identified the DEGs in glioblastoma multiforme using microarray data. An enrichment evaluation identified 10 hub genes that may serve as targets for treatments. The top-ranking gene with the highest level was discovered to be MMP9. The role of MMP9 in glioblastoma multiforme has been explored by different studies, and the expression of MMP9 was reported to be significantly upregulated in highly malignant gliomas and correlates with the progression, suggesting a role for MMP9 in promoting the observed invasiveness (Rao et al., 1996). The development of GBM is demonstrated to be significantly influenced by POSTN (Park et al., 2016). HES5 was identified as a possible target for therapeutic intervention due to its involvement in a number of cancer-related processes, including increased cell proliferation (Wang et al., 2021). The other targets we identified are crucial for the development of GBM. The importance of these hub genes was highlighted by enrichment analysis, subnetwork building, and the inclusion of all hub genes within these subnetworks. The roles these genes play in various interrelated pathways were also clarified through the examination of KEGG pathways, molecular functions, cellular components, and biological processes. Determining the regulatory functions of transcription factors has been made possible by the identification of these factors and their roles within the enlarged network. The importance of ECM–receptor interaction and Notch signaling pathways in GBM has also been mentioned in earlier studies. Our KEGG pathways in this study include these specific pathways as well. Additionally, according to our research, GBM patients have considerable changes in immune-related pathways (Kanderi and Gupta, 2023b).

Furthermore, the drugs we discovered for these targets have given us a useful insight into how to potentially inhibit these targets and find brand-new FDA-approved medicines.

Furthermore, survival analysis extensively cleared the role of these hub genes in the progression of breast cancer. Because the compliance of patients to adjuvant treatment is different, this may influence the treatment result. This bioinformatics model suggested a catalog of candidate cellular proteins that could be the targets for breast cancer therapy. An advance computational strategy has been created to investigate the FDA-approved drug molecules screened against the MMP9 hub gene. While performing computer-aided drug design, the ligand structure has been refined against the therapeutic target. Furthermore, for validation of the docked complexes, MD simulation in the drug design process has far-reaching implications to study the dynamic behavior of receptor–ligand complexes. Molecular docking methods alone fail to address structural variability by limiting receptor flexibility and reducing the freedom of ligand-bound conformations. A simulation approach was used to examine the dynamic behavior of MMP9, which offered valuable insights into the structural basis of its potential as a drug.

## 5 Conclusion

In conclusion, our study not only provides important new understandings of putative biomarkers linked to patient prognoses for glioblastoma multiforme tumors but also emphasizes how critical it is to assess transcription factors and PPI networks as a solid framework. Identifying potential biomarkers and gaining a thorough grasp of the fundamental aspects affecting the prognosis of GBM patients are the main contributions of our research. All four of the compounds were confirmed as possible MMP9 inhibitors with high binding scores using the molecular docking approach. Furthermore, due to structural rearrangements in a physiochemical environment, MD simulations showed the extraordinary stability of receptor complexes including temozolomide and lomustine over a 200-ns time scale compared to Apo-state and with a control inhibitor system. Interestingly, small alterations to the side chain and loop movement of these inhibitors showed very little effect on their stability. The potential of the chosen ligands as lead-like compounds was confirmed by the structural stability that was observed in the docked complexes after simulation. The binding energies finally calculated showed high binding affinities of the complexes throughout the time intervals. Using the strong insights obtained from this research, our study encourages researchers to investigate the creation of a modified and precisely targeted drug against GBM in light of these results. The continuous stability of the system throughout the simulations provides a solid basis for future research and development of successful glioblastoma multiforme therapies.

## Data Availability

The original contributions presented in the study are included in the article/Supplementary Material; further inquiries can be directed to the corresponding authors.
