# Supplementary material for: System biology approach to identify the novel biomarkers in glioblastoma multiforme tumors by using computational analysis
Source: Front Pharmacol. 2024 May 22;15:1364138. doi: 10.3389/fphar.2024.1364138 (PMC11150670; doi:10.3389/fphar.2024.1364138)
Supplement: Supplementary file 1 [file Table1.DOCX]

Supplementary Files

**System Biology Approach to Identify the Novel Biomarkers in Glioblastoma Multiform Tumors by Using Computational Analysis**

Safar M. Alqahtani^1,*^, Ali Altharawi^1^, Alhumaidi Alabbas^1^, Faisal Ahmad^2,3^, Hassan Ayaz^4^*,Asia Nawaz^4^, Sidra Rahman^4^, Manal A. Alossaimi^1^

^1^Department of Pharmaceutical Chemistry, College of Pharmacy, Prince Sattam Bin Abdulaziz University, Al Kharj 11942, Saudi Arabia; [safar.alqahtani@psau.edu.sa](mailto:safar.alqahtani@psau.edu.sa) (S.A.); [a.altharawi@psau.edu.sa](mailto:a.altharawi@psau.edu.sa) (A.Ai.); [ab.alabbas@psau.edu.sa](mailto:ab.alabbas@psau.edu.sa) (A.As.); [m.alossaimi@psau.edu.sa](mailto:m.alossaimi@psau.edu.sa) (M.A.A.).

^2^Foundation University Medical College, Foundation University Islamabad, Islamabad 44000, Pakistan, [faisalahmad@bs.qau.edu.pk](mailto:faisalahmad@bs.qau.edu.pk)

^3^School of Biology Georgia Institute of Technology, Atlanta, Georgia 30332-0230 USA, [fahmad43@gatech.edu](mailto:fahmad43@gatech.edu)

^4^Department of Biotechnology, Quaid-i-Azam University Islamabad, Pakistan, [hassanayaz133@gmail.com](mailto:hassanayaz133@gmail.com), [asiyanawazgcu@gmail.com](mailto:asiyanawazgcu@gmail.com), Sidrarahman1716@yahoo.com

***Correspondence**: [safar.alqahtani@psau.edu.sa](mailto:safar.alqahtani@psau.edu.sa) (S.A.); [hassanayaz133@gmail.com](mailto:hassanayaz133@gmail.com) (H.A)

**S-Table 1.** Showing the distribution of DEGs in highly significant cellular components, molecular functions and KEGG pathways based on FDR significant value.

| **GO ID** | **Term description** | | **Matching proteins in the network** | | **False discovery rate** | | | |  |
| --- | --- | --- | --- | --- | --- | --- | --- | --- | --- |
| **Go Cellular components** | | | | | | | | |  |
| GO:0005615 | extracellular space | | PCSK1, MOXD1, TNFAIP6, F13A1, FGF1, TFPI, NID2, CXCL14, LGALS3, SRPX2, CREG2, SPOCK3, EMILIN1, LEFTY2, TIMP4, CTHRC1, POSTN, MIA, PCOLCE, MMP9, DKK1, C1QL1, BCAN, BMP2, COL6A2, SMOC1, COL5A2, CHI3L1, LRRN1, ANGPTL4, LTF | | 1.04E-04 | | | |  |
| GO:0005576 | extracellular region | | TNFAIP6, F13A1, FGF1, TFPI, NID2, DLL1, GLB1L2, LGALS3, MDK, SPOCK3, EMILIN1, NPTX2, LEFTY2, CTHRC1, POSTN, SPAG11A, PCOLCE, NPNT, MMP9, DKK1, BCAN, BMP2, COL6A2, SMOC1, COL5A2, CHI3L1, CSPG5, ANGPTL4, LTF | | 0.003561705 | | | |  |
| GO:0031012 | extracellular matrix | | BCAN, POSTN, COL6A2, COL5A2, SPOCK3, CHI3L1, LRRN1, FGF1, TIMP4 | | 0.008083051 | | | |  |
| GO:0005581 | collagen trimer | | COL6A2, COL5A2, EMILIN1, PCOLCE, C1QL1, CTHRC1 | | 0.00967861 | | | |  |
| GO:0009986 | cell surface | | LGALS3, BMP2, EGFLAM, SRPX2, SDC4, CSPG5, CNTN2, ITGB7, CD24, TFPI, CACNG4, LTF | | 0.054210226 | | | |  |
| GO:0005604 | basement membrane | | EGFLAM, SMOC1, NPNT, NID2 | | 0.550208865 | | | |  |
| GO:0005891 | voltage-gated calcium channel complex | | SCN10A, SCN3A, CACNG4 | | 0.550208865 | | | |  |
| GO:0097427 | microtubule bundle | | TPPP3, MARK2 | | 0.763356091 | | | |  |
| GO:0045202 | synapse | | CAMK2B, GABRA2, BCAN, SRPX2, CSPG5, CNTN2, PCDHB10, PCDHB9 | | 0.763356091 | | | |  |
| GO:1904724 | tertiary granule lumen | | TNFAIP6, MMP9, LTF | | 0.763784287 | | | |  |
| GO:0048471 | perinuclear region of cytoplasm | | TMEM100, PCSK1, TPPP3, SLC2A10, RAB34, ATN1, CHI3L1, SLC39A12, MYO16 | | 1 | | | |  |
| GO:0098982 | GABA-ergic synapse | | GABRA2, KCND2, CSPG5 | | 1 | | | |  |
| GO:0098978 | glutamatergic synapse | | BCAN, SCN10A, KCND2, CSPG5, NPTX2, CACNG4 | | 1 | | | |  |
| GO:0016528 | sarcoplasm | | SCN3A, CTHRC1 | | 1 | | | |  |
| GO:0001518 | voltage-gated sodium channel complex | | SCN10A, SCN3A | | 1 | | | |  |
| **GO ID** | **Term description** | | | **Matching proteins in the network** | | **False discovery rate** | | | |
| **Go molecular function** |  |  |  |  |  |  |  |  |  |
| GO:0005201 | extracellular matrix structural constituent | | | POSTN, SRPX2, COL5A2, CHI3L1, EMILIN1, PCOLCE, NPNT, NID2, CTHRC1 | | 4.23E-04 | | | |
| GO:0008083 | growth factor activity | | | BMP2, MIA, MDK, CSPG5, FGF1, LEFTY2, DKK1 | | 0.061715 | | | |
| GO:0008201 | heparin binding | | | POSTN, MDK, SMOC1, PCOLCE, FGF1, LTF | | 0.35825 | | | |
| GO:0030246 | carbohydrate binding | | | BCAN, LGALS3, KLRC3, CNTN2, CHI3L1, NPTX2 | | 0.380758 | | | |
| GO:0005518 | collagen binding | | | COL6A2, PCOLCE, NID2, MMP9 | | 0.380758 | | | |
| GO:0005509 | calcium ion binding | | | NECAB1, EGFLAM, TNFAIP6, SMOC1, SPOCK3, NPNT, DLL1, NID2, PCDHB10, PCDHB9, DLL3 | | 0.581093 | | | |
| GO:1990837 | sequence-specific double-stranded DNA binding | | | IRX1, HES6, ZNF384, MAFG, OLIG2, NR2E1, ASCL1, MEOX2, HES5 | | 0.664792 | | | |
| GO:0042802 | identical protein binding | | | CAMK2B, PCSK1, NECAB1, MAOB, SDC4, CISD1, OLIG2, ASCL1, MMP9, SRPX2, MAFG, MKRN3, CNTN2, EMILIN1, ANGPTL4, ASPA, GAS7 | | 1 | | | |
| GO:0033300 | dehydroascorbic acid transporter activity | | | SLC2A10, SLC2A6 | | 1 | | | |
| GO:0016655 | oxidoreductase activity, acting on NAD(P)H, quinone or similar compound as acceptor | | | CBR1, AKR1C1 | | 1 | | | |
| GO:0005178 | integrin binding | | | FERMT1, ITGB7, FGF1, NPNT | | 1 | | | |
| GO:0055056 | D-glucose transmembrane transporter activity | | | SLC2A10, SLC2A6 | | 1 | | | |
| GO:0039706 | co-receptor binding | | | BMP2, DKK1 | | 1 | | | |
| GO:0008191 | metalloendopeptidase inhibitor activity | | | SPOCK3, TIMP4 | | 1 | | | |
| GO:0046983 | protein dimerization activity | | | HES6, OLIG2, ASCL1, HES5 | | 1 | | | |
| **GO ID** | **Term description** | **Matching proteins in the network** | | | | | **False discovery rate** |  |  |
| **KEGG Pathways** |  |  |  |  |  |  |  |  |  |
| hsa04512 | ECM-receptor interaction | SDC4, COL6A2, ITGB7, NPNT | | | | | 1 |  |  |
| hsa04330 | Notch signaling pathway | DLL1, HES5, DLL3 | | | | | 1 |  |  |
| hsa05224 | Breast cancer | FGF1, DLL1, HES5, DLL3 | | | | | 1 |  |  |
| hsa05200 | Pathways in cancer | CAMK2B, BMP2, FGF1, DLL1, MMP9, HES5, DLL3 | | | | | 1 |  |  |
| hsa01522 | Endocrine resistance | DLL1, MMP9, DLL3 | | | | | 1 |  |  |

**S-Table 2**. Showing the distribution of DEGs in highly significant biological processes based on FDR significant value.

| **GO ID** | **Term description** | **Matching proteins in the network** | **False discovery rate** |
| --- | --- | --- | --- |
| **Go biological processes** | | | |
| GO:0030198 | extracellular matrix organization | CSGALNACT1, POSTN, EGFLAM, MIA, SMOC1, COL5A2, NR2E1, NPNT, MMP9 | 0.013532 |
| GO:0007155 | cell adhesion | POSTN, TNFAIP6, NPNT, NID2, PCDHB10, BCAN, FERMT1, COL6A2, CNTN2, EMILIN1, ITGB7, CD24, PCDHB9, HES5 | 0.013532 |
| GO:0045665 | negative regulation of neuron differentiation | CNTN2, OLIG2, NR2E1, ASCL1, DLL1, HES5 | 0.013532 |
| GO:0007219 | Notch signaling pathway | TMEM100, BMP2, ASCL1, DLL1, TIMP4, HES5, DLL3 | 0.019292 |
| GO:0045669 | positive regulation of osteoblast differentiation | BMP2, SOX11, NPNT, CTHRC1, LTF | 0.212721 |
| GO:0001501 | skeletal system development | BCAN, BMP2, COL5A2, SOX11, MMP9, DLL3 | 0.212721 |
| GO:2000726 | negative regulation of cardiac muscle cell differentiation | BMP2, DLL1, DKK1 | 0.22069 |
| GO:0050767 | regulation of neurogenesis | HES6, ASCL1, DLL1, HES5 | 0.22069 |
| GO:0033690 | positive regulation of osteoblast proliferation | BMP2, CTHRC1, LTF | 0.296773 |
| GO:0022010 | central nervous system myelination | CNTN2, ASPA, HES5 | 0.356757 |
| GO:0007160 | cell-matrix adhesion | FERMT1, EMILIN1, ITGB7, NPNT, NID2 | 0.381789 |
| GO:0048708 | astrocyte differentiation | BMP2, NR2E1, HES5 | 0.422071 |
| GO:0016477 | cell migration | SDC4, ATN1, EMILIN1, ITGB7, CD24, MMP9, CTHRC1 | 0.422071 |
| GO:0060122 | inner ear receptor stereocilium organization | SDC4, HES5, CTHRC1 | 0.422071 |
| GO:2000179 | positive regulation of neural precursor cell proliferation | MDK, NR2E1, ASCL1 | 0.422071 |
| GO:0014003 | oligodendrocyte development | SOX11, ASCL1, HES5 | 0.520783 |
| GO:0048714 | positive regulation of oligodendrocyte differentiation | MDK, OLIG2, ASPA | 0.622345 |
| GO:1903672 | positive regulation of sprouting angiogenesis | SLC39A12, FGF1, DLL1 | 0.67834 |
| GO:0007399 | nervous system development | CAMK2B, GPSM1, HES6, MDK, CSPG5, SOX11, NR2E1, PCDHB9 | 0.680707 |
| GO:0045766 | positive regulation of angiogenesis | CHI3L1, EMILIN1, ANGPTL4, NR2E1, FGF1 | 0.71667 |
| GO:0030511 | positive regulation of transforming growth factor beta receptor signaling pathway | FERMT1, SLC2A10, NPNT | 0.71667 |
| GO:1900159 | positive regulation of bone mineralization involved in bone maturation | BMP2, LTF | 0.71667 |
| GO:0021530 | spinal cord oligodendrocyte cell fate specification | OLIG2, ASCL1 | 0.71667 |
| GO:0019228 | neuronal action potential | SCN10A, KCND2, SCN3A | 0.71667 |
| GO:0001525 | angiogenesis | TMEM100, SRPX2, ANGPTL4, NR2E1, FGF1, MEOX2 | 0.71667 |
| GO:0090090 | negative regulation of canonical Wnt signaling pathway | FERMT1, BMP2, MDK, DKK1, CTHRC1 | 0.71667 |
| GO:0010811 | positive regulation of cell-substrate adhesion | EGFLAM, EMILIN1, NPNT | 0.71667 |
| GO:0030509 | BMP signaling pathway | TMEM100, BMP2, LEFTY2, HES5 | 0.71667 |
| GO:0030154 | cell differentiation | CAMK2B, GPSM1, HES6, MDK, SMOC1, SOX11, NR2E1, FGF1, DLL1, DLL3 | 0.725019 |
| GO:0060173 | limb development | SMOC1, MEOX2, DKK1 | 0.725019 |
| GO:0007386 | compartment pattern specification | DLL1, DLL3 | 0.725019 |
| GO:0072086 | specification of loop of Henle identity | IRX1, HES5 | 0.725019 |
| GO:0045608 | negative regulation of auditory receptor cell differentiation | DLL1, HES5 | 0.725019 |
| GO:0061844 | antimicrobial humoral immune response mediated by antimicrobial peptide | LGALS3, SPAG11A, CXCL14, LTF | 0.82047 |
| GO:0001757 | somite specification | MEOX2, DLL1 | 0.831866 |
| GO:0045747 | positive regulation of Notch signaling pathway | ASCL1, DLL1, HES5 | 0.831866 |
| GO:0048839 | inner ear development | BMP2, DLL1, CXCL14 | 0.886922 |
| GO:0010628 | positive regulation of gene expression | BMP2, SLC2A10, MAFG, SOX11, EMILIN1, FGF1, DLL1, DKK1 | 0.886922 |
| GO:0000122 | negative regulation of transcription from RNA polymerase II promoter | IRX1, HES6, BMP2, MAFG, ATN1, SOX11, OLIG2, NR2E1, DUSP26, ASCL1, DKK1, HES5 | 0.886922 |
| GO:0019800 | peptide cross-linking via chondroitin 4-sulfate glycosaminoglycan | EGFLAM, SPOCK3 | 0.891922 |
| GO:0001841 | neural tube formation | SOX11, SLC39A12 | 0.999011 |
| GO:0030856 | regulation of epithelial cell differentiation | CD24, ASCL1 | 0.999011 |
| GO:0061743 | motor learning | DKK1, C1QL1 | 0.999011 |
| GO:0000226 | microtubule cytoskeleton organization | CCDC8, TACC3, CNTN2, MARK2 | 0.999011 |
| GO:0009887 | animal organ morphogenesis | BMP2, EGFLAM, MEIS3P1, FGF1 | 0.999011 |
| GO:0070837 | dehydroascorbic acid transport | SLC2A10, SLC2A6 | 0.999011 |
| GO:0051216 | cartilage development | CSGALNACT1, CHI3L1, HES5 | 0.999011 |
| GO:0042420 | dopamine catabolic process | MOXD1, MAOB | 0.999011 |
| GO:0045944 | positive regulation of transcription from RNA polymerase II promoter | ZNF384, SOX11, OLIG2, NR2E1, FGF1, ASCL1, NPNT, MEOX2, DLL1, BMP2, MAFG, MEIS3P1, HES5 | 0.999011 |
| GO:0060563 | neuroepithelial cell differentiation | SOX11, DLL1 | 0.999011 |
| GO:2000347 | positive regulation of hepatocyte proliferation | MDK, FGF1 | 0.999011 |
| GO:0048712 | negative regulation of astrocyte differentiation | NR2E1, HES5 | 0.999011 |
| GO:0007268 | chemical synaptic transmission | GABRA2, KCND2, NPTX2, PCDHB10, PCDHB9 | 0.999011 |
| GO:0008645 | hexose transport | SLC2A10, SLC2A6 | 0.999011 |
| GO:0030335 | positive regulation of cell migration | BMP2, TNFAIP6, MDK, FGF1, MMP9 | 0.999011 |
| GO:0021987 | cerebral cortex development | MDK, TACC3, ASCL1 | 0.999011 |
| GO:0060394 | negative regulation of pathway-restricted SMAD protein phosphorylation | EMILIN1, DKK1 | 0.999011 |
| GO:0030182 | neuron differentiation | IRX1, SOX11, OLIG2, ASCL1 | 0.999011 |
| GO:0032966 | negative regulation of collagen biosynthetic process | ERRFI1, EMILIN1 | 0.999011 |
| GO:0042491 | auditory receptor cell differentiation | DLL1, HES5 | 0.999011 |
| GO:0045778 | positive regulation of ossification | BMP2, SOX11 | 0.999011 |
| GO:0001503 | ossification | COL5A2, MMP9, LTF | 0.999011 |
| GO:0070373 | negative regulation of ERK1 and ERK2 cascade | ERRFI1, EMILIN1, DUSP26 | 0.999011 |
| GO:0061053 | somite development | SOX11, MEOX2 | 0.999011 |
| GO:0090162 | establishment of epithelial cell polarity | FERMT1, HES5 | 0.999011 |
| GO:0086010 | membrane depolarization during action potential | SCN10A, SCN3A | 0.999011 |
| GO:0048485 | sympathetic nervous system development | SOX11, ASCL1 | 0.999011 |
| GO:0042325 | regulation of phosphorylation | SRPX2, CD24 | 0.999011 |
| GO:0043066 | negative regulation of apoptotic process | PLK2, ANGPTL4, NR2E1, ASCL1, MMP9, DKK1, LTF | 0.999011 |
| GO:0021537 | telencephalon development | BMP2, HES5 | 0.999011 |
| GO:0061036 | positive regulation of cartilage development | BMP2, MDK | 0.999011 |
