# Supplementary material for: System biology approach to identify the novel biomarkers in glioblastoma multiforme tumors by using computational analysis
Source: Front Pharmacol. 2024 May 22;15:1364138. doi: 10.3389/fphar.2024.1364138 (PMC11150670; doi:10.3389/fphar.2024.1364138)
Supplement: Supplementary file 3 [file Table2.docx]

**Tables S3.** TF and Kinases identification with hypergeometric p-value and Z-score.

| **Results** | | | | |
| --- | --- | --- | --- | --- |
| **Rank** | **Transcription Factor** | **Hypergeometric p-value** | **Z-score** | **Combined score** |
| 1 | *SUZ12* | 5.990e-11 | 0.00 | 0.00 |
| 2 | *EZH2* | 0.004006 | 0.00 | 0.00 |
| 3 | *TRIM28* | 0.01082 | 0.00 | 0.00 |
| 4 | *SOX2* | 0.05378 | 0.00 | 0.00 |
| 5 | *REST* | 0.05549 | 0.00 | 0.00 |
| 6 | *SMAD4* | 0.07392 | 0.00 | 0.00 |
| 7 | *NANOG* | 0.07976 | 0.00 | 0.00 |
| 8 | *AR* | 0.07999 | 0.00 | 0.00 |
| 9 | *RAD21* | 0.0957 | 0.00 | 0.00 |
| 10 | *PPARD* | 0.1047 | 0.00 | 0.00 |
| 11 | *EZH2* | 0.1625 | 0.00 | 0.00 |
| 12 | *SALL4* | 0.1831 | 0.00 | 0.00 |
| 13 | *SMC3* | 0.2018 | 0.00 | 0.00 |
| 14 | *POU5F1* | 0.2247 | 0.00 | 0.00 |
| 15 | *IRF1* | 0.2279 | 0.00 | 0.00 |
| 16 | *MYOD1* | 0.2793 | 0.00 | 0.00 |
| 17 | *RUNX1* | 0.2874 | 0.00 | 0.00 |
| 18 | *TCF3* | 0.2916 | 0.00 | 0.00 |
| 19 | *EGR1* | 0.315 | 0.00 | 0.00 |
| 20 | *CBX3* | 0.4 | 0.00 | 0.00 |
| 21 | *KLF4* | 0.4198 | 0.00 | 0.00 |
| 22 | *PPARG* | 0.4297 | 0.00 | 0.00 |
| 23 | *NELFE* | 0.431 | 0.00 | 0.00 |
| 24 | *FOXM1* | 0.4503 | 0.00 | 0.00 |
| 25 | *MYC* | 0.482 | 0.00 | 0.00 |
| 26 | *SUZ12* | 0.4836 | 0.00 | 0.00 |
| 27 | *CEBPD* | 0.4855 | 0.00 | 0.00 |
| 28 | *ZEB1* | 0.4868 | 0.00 | 0.00 |
| 29 | *TCF7L2* | 0.4954 | 0.00 | 0.00 |
| 30 | *TRIM28* | 0.5299 | 0.00 | 0.00 |
| 31 | *GATA2* | 0.5303 | 0.00 | 0.00 |
| 32 | *NFE2L2* | 0.6191 | 0.00 | 0.00 |
| 33 | *ESR1* | 0.6201 | 0.00 | 0.00 |
| 34 | *REST* | 0.693 | 0.00 | 0.00 |
| 35 | *FOSL2* | 0.7083 | 0.00 | 0.00 |
| 36 | *GATA1* | 0.7466 | 0.00 | 0.00 |
| 37 | *TP63* | 0.779 | 0.00 | 0.00 |
| 38 | *CHD1* | 0.78 | 0.00 | 0.00 |
| 39 | *CTCF* | 0.7977 | 0.00 | 0.00 |
| 40 | *RELA* | 0.808 | 0.00 | 0.00 |
| 41 | *BCL3* | 0.8205 | 0.00 | 0.00 |
| 42 | *NFIC* | 0.8272 | 0.00 | 0.00 |
| 43 | *ZNF384* | 0.8384 | 0.00 | 0.00 |
| 44 | *SPI1* | 0.8557 | 0.00 | 0.00 |
| 45 | *USF2* | 0.8583 | 0.00 | 0.00 |
| 46 | *FOXA2* | 0.8633 | 0.00 | 0.00 |
| 47 | *TP53* | 0.8659 | 0.00 | 0.00 |
| 48 | *BCLAF1* | 0.9048 | 0.00 | 0.00 |
| 49 | *FOS* | 0.9106 | 0.00 | 0.00 |
| 50 | *TAF7* | 0.912 | 0.00 | 0.00 |
| 51 | *ZMIZ1* | 0.9287 | 0.00 | 0.00 |
| 52 | *ZBTB33* | 0.9363 | 0.00 | 0.00 |
| 53 | *RCOR1* | 0.9363 | 0.00 | 0.00 |
| 54 | *MAX* | 0.9549 | 0.00 | 0.00 |
| 55 | *HNF4A* | 0.9619 | 0.00 | 0.00 |
| 56 | *EGR1* | 0.9667 | 0.00 | 0.00 |
| 57 | *TCF3* | 0.9698 | 0.00 | 0.00 |
| 58 | *E2F1* | 0.9728 | 0.00 | 0.00 |
| 59 | *SIN3A* | 0.9751 | 0.00 | 0.00 |
| 60 | *USF1* | 0.9808 | 0.00 | 0.00 |
| 61 | *FLI1* | 0.9839 | 0.00 | 0.00 |
| 62 | *IRF3* | 0.9852 | 0.00 | 0.00 |
| 63 | *SP1* | 0.9889 | 0.00 | 0.00 |
| 64 | *E2F4* | 0.9891 | 0.00 | 0.00 |
| 65 | *SPI1* | 0.9911 | 0.00 | 0.00 |
| 66 | *SIX5* | 0.9928 | 0.00 | 0.00 |
| 67 | *NRF1* | 0.9935 | 0.00 | 0.00 |
| 68 | *CREB1* | 0.9952 | 0.00 | 0.00 |
| 69 | *PBX3* | 0.9975 | 0.00 | 0.00 |
| 70 | *E2F6* | 0.998 | 0.00 | 0.00 |
| 71 | *SP2* | 0.9983 | 0.00 | 0.00 |
| 72 | *UBTF* | 0.9983 | 0.00 | 0.00 |
| 73 | *NFYB* | 0.9985 | 0.00 | 0.00 |
| 74 | *ZBTB7A* | 0.9986 | 0.00 | 0.00 |
| 75 | *MYC* | 0.9994 | 0.00 | 0.00 |
| 76 | *NFYA* | 0.9998 | 0.00 | 0.00 |
| 77 | *CREB1* | 1 | 0.00 | 0.00 |
| 78 | *PML* | 1 | 0.00 | 0.00 |
| 79 | *GABPA* | 1 | 0.00 | 0.00 |
| 80 | *ATF2* | 1 | 0.00 | 0.00 |
| 81 | *YY1* | 1 | 0.00 | 0.00 |
| 82 | *ELF1* | 1 | 0.00 | 0.00 |
| 83 | *BRCA1* | 1 | 0.00 | 0.00 |
| 84 | *TAF1* | 1 | 0.00 | 0.00 |

| **Results** | | | | |
| --- | --- | --- | --- | --- |
| **Rank** | **Protein Kinase** | **Hypergeometric p-value** | **Z-score** | **Combined score** |
| 1 | *CDK1* | 3.928e-18 | 0.00 | 0.00 |
| 2 | *CSNK2A1* | 6.992e-16 | 0.00 | 0.00 |
| 3 | *MAPK14* | 3.662e-14 | 0.00 | 0.00 |
| 4 | *CDK2* | 2.945e-13 | 0.00 | 0.00 |
| 5 | *GSK3B* | 3.961e-12 | 0.00 | 0.00 |
| 6 | *HIPK2* | 8.716e-12 | 0.00 | 0.00 |
| 7 | *MAPK1* | 2.648e-11 | 0.00 | 0.00 |
| 8 | *MAPK3* | 3.298e-10 | 0.00 | 0.00 |
| 9 | *AKT1* | 5.608e-10 | 0.00 | 0.00 |
| 10 | *CDK4* | 9.105e-10 | 0.00 | 0.00 |
| 11 | *ERK2* | 4.017e-9 | 0.00 | 0.00 |
| 12 | *ERK1* | 6.018e-8 | 0.00 | 0.00 |
| 13 | *CK2ALPHA* | 1.051e-7 | 0.00 | 0.00 |
| 14 | *GSK3BETA* | 1.495e-7 | 0.00 | 0.00 |
| 15 | *IKKALPHA* | 6.205e-7 | 0.00 | 0.00 |
| 16 | *CDK8* | 0.00000168 | 0.00 | 0.00 |
| 17 | *TGFBR2* | 0.000002586 | 0.00 | 0.00 |
| 18 | *ATM* | 0.000004805 | 0.00 | 0.00 |
| 19 | *JNK1* | 0.000009466 | 0.00 | 0.00 |
| 20 | *PKBALPHA* | 0.00003791 | 0.00 | 0.00 |
| 21 | *DNAPK* | 0.00004091 | 0.00 | 0.00 |
| 22 | *PIM1* | 0.00004504 | 0.00 | 0.00 |
| 23 | *ERBB2* | 0.00004627 | 0.00 | 0.00 |
| 24 | *PRKCZ* | 0.00005897 | 0.00 | 0.00 |
| 25 | *MAPK8* | 0.00007073 | 0.00 | 0.00 |
| 26 | *CSNK1E* | 0.00009289 | 0.00 | 0.00 |
| 27 | *RPS6KA5* | 0.0001459 | 0.00 | 0.00 |
| 28 | *PKR* | 0.0001619 | 0.00 | 0.00 |
| 29 | *NLK* | 0.0002178 | 0.00 | 0.00 |
| 30 | *JAK2* | 0.0004049 | 0.00 | 0.00 |
| 31 | *CDC2* | 0.0004141 | 0.00 | 0.00 |
| 32 | *CHUK* | 0.0004724 | 0.00 | 0.00 |
| 33 | *CDK9* | 0.0007927 | 0.00 | 0.00 |
| 34 | *CHEK1* | 0.0008755 | 0.00 | 0.00 |
| 35 | *PRKCD* | 0.001408 | 0.00 | 0.00 |
| 36 | *RPS6KA3* | 0.00171 | 0.00 | 0.00 |
| 37 | *PRKDC* | 0.001788 | 0.00 | 0.00 |
| 38 | *CSNK1D* | 0.002073 | 0.00 | 0.00 |
| 39 | *IKKBETA* | 0.002234 | 0.00 | 0.00 |
| 40 | *TBK1* | 0.00236 | 0.00 | 0.00 |
| 41 | *IKK* | 0.00248 | 0.00 | 0.00 |
| 42 | *IKBKE* | 0.002486 | 0.00 | 0.00 |
| 43 | *PRKACA* | 0.002679 | 0.00 | 0.00 |
| 44 | *CHEK2* | 0.00277 | 0.00 | 0.00 |
| 45 | *ABL1* | 0.003151 | 0.00 | 0.00 |
| 46 | *GSK* | 0.004781 | 0.00 | 0.00 |
| 47 | *UHMK1* | 0.005234 | 0.00 | 0.00 |
| 48 | *MAP3K7* | 0.005677 | 0.00 | 0.00 |
| 49 | *CDK5* | 0.005718 | 0.00 | 0.00 |
| 50 | *CK2A2* | 0.006111 | 0.00 | 0.00 |
| 51 | *SRC* | 0.007068 | 0.00 | 0.00 |
| 52 | *CAMKIV* | 0.007517 | 0.00 | 0.00 |
| 53 | *CK2* | 0.0079 | 0.00 | 0.00 |
| 54 | *PDHK1* | 0.009123 | 0.00 | 0.00 |
| 55 | *RET* | 0.009781 | 0.00 | 0.00 |
| 56 | *ALK* | 0.009781 | 0.00 | 0.00 |
| 57 | *CDK7* | 0.01143 | 0.00 | 0.00 |
| 58 | *MAPK11* | 0.01299 | 0.00 | 0.00 |
| 59 | *ACTR2B* | 0.01328 | 0.00 | 0.00 |
| 60 | *PRKCA* | 0.01457 | 0.00 | 0.00 |
| 61 | *CSNK1A1* | 0.01464 | 0.00 | 0.00 |
| 62 | *AURORAA* | 0.01523 | 0.00 | 0.00 |
| 63 | *RAF1* | 0.01756 | 0.00 | 0.00 |
| 64 | *PKBBETA* | 0.01792 | 0.00 | 0.00 |
| 65 | *MAP2K3* | 0.01889 | 0.00 | 0.00 |
| 66 | *GSK3A* | 0.02092 | 0.00 | 0.00 |
| 67 | *EGFR* | 0.02173 | 0.00 | 0.00 |
| 68 | *GSK3ALPHA* | 0.02197 | 0.00 | 0.00 |
| 69 | *FOXO3* | 0.02213 | 0.00 | 0.00 |
| 70 | *TRIM33* | 0.02213 | 0.00 | 0.00 |
| 71 | *HASPIN* | 0.02213 | 0.00 | 0.00 |
| 72 | *TGM2* | 0.02213 | 0.00 | 0.00 |
| 73 | *LYK5* | 0.02213 | 0.00 | 0.00 |
| 74 | *PIKFYVE* | 0.02213 | 0.00 | 0.00 |
| 75 | *ROR1* | 0.02242 | 0.00 | 0.00 |
| 76 | *LRRK1* | 0.02242 | 0.00 | 0.00 |
| 77 | *ACVR2B* | 0.02242 | 0.00 | 0.00 |
| 78 | *PKA* | 0.02448 | 0.00 | 0.00 |
| 79 | *PIM3* | 0.02662 | 0.00 | 0.00 |
| 80 | *GSG2* | 0.02662 | 0.00 | 0.00 |
| 81 | *ERBB3* | 0.03017 | 0.00 | 0.00 |
| 82 | *PRKCB* | 0.03105 | 0.00 | 0.00 |
| 83 | *MERTK* | 0.03112 | 0.00 | 0.00 |
| 84 | *DYRK3* | 0.03112 | 0.00 | 0.00 |
| 85 | *TGFBR1* | 0.03277 | 0.00 | 0.00 |
| 86 | *PBK* | 0.03347 | 0.00 | 0.00 |
| 87 | *RPS6KA4* | 0.03347 | 0.00 | 0.00 |
| 88 | *LYN* | 0.03412 | 0.00 | 0.00 |
| 89 | *PIM2* | 0.0359 | 0.00 | 0.00 |
| 90 | *EPHA8* | 0.0359 | 0.00 | 0.00 |
| 91 | *ILK* | 0.03838 | 0.00 | 0.00 |
| 92 | *MELK* | 0.04093 | 0.00 | 0.00 |
| 93 | *PKBGAMMA* | 0.04355 | 0.00 | 0.00 |
| 94 | *TNK2* | 0.04355 | 0.00 | 0.00 |
| 95 | *MAP3K20* | 0.04378 | 0.00 | 0.00 |
| 96 | *JAK3* | 0.04622 | 0.00 | 0.00 |
| 97 | *PAK1* | 0.04674 | 0.00 | 0.00 |
| 98 | *PRKACB* | 0.04895 | 0.00 | 0.00 |
| 99 | *JAK1* | 0.04895 | 0.00 | 0.00 |
| 100 | *CAMK2D* | 0.04895 | 0.00 | 0.00 |
| 101 | *ATR* | 0.05153 | 0.00 | 0.00 |
| 102 | *AXL* | 0.05174 | 0.00 | 0.00 |
| 103 | *FER* | 0.05174 | 0.00 | 0.00 |
| 104 | *NPM/ALK* | 0.05443 | 0.00 | 0.00 |
| 105 | *BMPR1B* | 0.05749 | 0.00 | 0.00 |
| 106 | *PAK6* | 0.06344 | 0.00 | 0.00 |
| 107 | *RPS6KA1* | 0.06483 | 0.00 | 0.00 |
| 108 | *CAM* | 0.06496 | 0.00 | 0.00 |
| 109 | *PKM* | 0.06496 | 0.00 | 0.00 |
| 110 | *PKG1CGKI* | 0.0665 | 0.00 | 0.00 |
| 111 | *PKCEPSILON* | 0.06671 | 0.00 | 0.00 |
| 112 | *FYN* | 0.06934 | 0.00 | 0.00 |
| 113 | *AURKA* | 0.07054 | 0.00 | 0.00 |
| 114 | *MARK2* | 0.07274 | 0.00 | 0.00 |
| 115 | *CHK2* | 0.07537 | 0.00 | 0.00 |
| 116 | *PDHK2* | 0.07537 | 0.00 | 0.00 |
| 117 | *NIK* | 0.07537 | 0.00 | 0.00 |
| 118 | *DYRK2* | 0.07594 | 0.00 | 0.00 |
| 119 | *CSNK2A2* | 0.07631 | 0.00 | 0.00 |
| 120 | *DYRK1A* | 0.08246 | 0.00 | 0.00 |
| 121 | *IKBKB* | 0.08262 | 0.00 | 0.00 |
| 122 | *RSK* | 0.08567 | 0.00 | 0.00 |
| 123 | *RSK3* | 0.08567 | 0.00 | 0.00 |
| 124 | *PKCIOTA* | 0.09254 | 0.00 | 0.00 |
| 125 | *DNA* | 0.09585 | 0.00 | 0.00 |
| 126 | *PTK2* | 0.09945 | 0.00 | 0.00 |
| 127 | *TRRAP* | 0.1059 | 0.00 | 0.00 |
| 128 | *CK1GAMMA1* | 0.1059 | 0.00 | 0.00 |
| 129 | *PRKCI* | 0.1065 | 0.00 | 0.00 |
| 130 | *TTK* | 0.1139 | 0.00 | 0.00 |
| 131 | *PKC* | 0.1159 | 0.00 | 0.00 |
| 132 | *CK1* | 0.1159 | 0.00 | 0.00 |
| 133 | *MAP2K4* | 0.1173 | 0.00 | 0.00 |
| 134 | *PKCTHETA* | 0.1173 | 0.00 | 0.00 |
| 135 | *PRKAA1* | 0.1212 | 0.00 | 0.00 |
| 136 | *PDGFRALPHA* | 0.1355 | 0.00 | 0.00 |
| 137 | *BRD2* | 0.1355 | 0.00 | 0.00 |
| 138 | *MTOR* | 0.1361 | 0.00 | 0.00 |
| 139 | *PKAALPHA* | 0.1387 | 0.00 | 0.00 |
| 140 | *PKAGAMMA* | 0.1436 | 0.00 | 0.00 |
| 141 | *P70S6K* | 0.1451 | 0.00 | 0.00 |
| 142 | *PKC* | 0.1641 | 0.00 | 0.00 |
| 143 | *MAP3K8* | 0.1709 | 0.00 | 0.00 |
| 144 | *SGK1* | 0.1709 | 0.00 | 0.00 |
| 145 | *PRKG1* | 0.1749 | 0.00 | 0.00 |
| 146 | *MAPKAPK2* | 0.1788 | 0.00 | 0.00 |
| 147 | *BTK* | 0.1788 | 0.00 | 0.00 |
| 148 | *TAOK3* | 0.1917 | 0.00 | 0.00 |
| 149 | *MAST1* | 0.1917 | 0.00 | 0.00 |
| 150 | *POMK* | 0.1917 | 0.00 | 0.00 |
| 151 | *MAP2K1* | 0.199 | 0.00 | 0.00 |
| 152 | *PDK* | 0.2007 | 0.00 | 0.00 |
| 153 | *CDK12* | 0.2007 | 0.00 | 0.00 |
| 154 | *PKCETA* | 0.2007 | 0.00 | 0.00 |
| 155 | *MAST4* | 0.2007 | 0.00 | 0.00 |
| 156 | *PRKD1* | 0.203 | 0.00 | 0.00 |
| 157 | *TYK2* | 0.203 | 0.00 | 0.00 |
| 158 | *TYRO3* | 0.2097 | 0.00 | 0.00 |
| 159 | *DMPK* | 0.2097 | 0.00 | 0.00 |
| 160 | *PKMYT1* | 0.2097 | 0.00 | 0.00 |
| 161 | *CSNK2B* | 0.2097 | 0.00 | 0.00 |
| 162 | *PRKX* | 0.2097 | 0.00 | 0.00 |
| 163 | *LMTK2* | 0.2097 | 0.00 | 0.00 |
| 164 | *MARK4* | 0.2097 | 0.00 | 0.00 |
| 165 | *TRIB3* | 0.2185 | 0.00 | 0.00 |
| 166 | *PNCK* | 0.2185 | 0.00 | 0.00 |
| 167 | *BARK1* | 0.2194 | 0.00 | 0.00 |
| 168 | *INSR* | 0.2235 | 0.00 | 0.00 |
| 169 | *HIPK1* | 0.2272 | 0.00 | 0.00 |
| 170 | *TLK1* | 0.2272 | 0.00 | 0.00 |
| 171 | *SIK1* | 0.2272 | 0.00 | 0.00 |
| 172 | *CAMKK2* | 0.2272 | 0.00 | 0.00 |
| 173 | *MAP4K4* | 0.2272 | 0.00 | 0.00 |
| 174 | *MAPK6* | 0.2272 | 0.00 | 0.00 |
| 175 | *WEE1* | 0.2272 | 0.00 | 0.00 |
| 176 | *PRKCE* | 0.2317 | 0.00 | 0.00 |
| 177 | *CDK16* | 0.2358 | 0.00 | 0.00 |
| 178 | *CSNK1G1* | 0.2358 | 0.00 | 0.00 |
| 179 | *AURKC* | 0.2358 | 0.00 | 0.00 |
| 180 | *CAMK2G* | 0.2358 | 0.00 | 0.00 |
| 181 | *CAMKK1* | 0.2443 | 0.00 | 0.00 |
| 182 | *GRK5* | 0.2443 | 0.00 | 0.00 |
| 183 | *NEK1* | 0.2528 | 0.00 | 0.00 |
| 184 | *EPHB3* | 0.2528 | 0.00 | 0.00 |
| 185 | *MARK3* | 0.2528 | 0.00 | 0.00 |
| 186 | *AURKB* | 0.2542 | 0.00 | 0.00 |
| 187 | *VRK1* | 0.2611 | 0.00 | 0.00 |
| 188 | *ACVR1* | 0.2694 | 0.00 | 0.00 |
| 189 | *CK1EPSILON* | 0.2694 | 0.00 | 0.00 |
| 190 | *CLK2* | 0.2694 | 0.00 | 0.00 |
| 191 | *FGFR3* | 0.2694 | 0.00 | 0.00 |
| 192 | *IRAK1* | 0.2775 | 0.00 | 0.00 |
| 193 | *DYRK1B* | 0.2856 | 0.00 | 0.00 |
| 194 | *CDK6* | 0.2856 | 0.00 | 0.00 |
| 195 | *EPHA1* | 0.2856 | 0.00 | 0.00 |
| 196 | *PTK6* | 0.2856 | 0.00 | 0.00 |
| 197 | *CAMK4* | 0.2856 | 0.00 | 0.00 |
| 198 | *STK3* | 0.2856 | 0.00 | 0.00 |
| 199 | *MAPK7* | 0.2856 | 0.00 | 0.00 |
| 200 | *PTK2B* | 0.2856 | 0.00 | 0.00 |
| 201 | *LCK* | 0.2905 | 0.00 | 0.00 |
| 202 | *PDK1* | 0.2936 | 0.00 | 0.00 |
| 203 | *LATS2* | 0.2936 | 0.00 | 0.00 |
| 204 | *TEC* | 0.2936 | 0.00 | 0.00 |
| 205 | *PKN1* | 0.3015 | 0.00 | 0.00 |
| 206 | *NEK6* | 0.3015 | 0.00 | 0.00 |
| 207 | *CDK3* | 0.3015 | 0.00 | 0.00 |
| 208 | *LRRK2* | 0.3015 | 0.00 | 0.00 |
| 209 | *JNK2* | 0.3015 | 0.00 | 0.00 |
| 210 | *EPHB1* | 0.317 | 0.00 | 0.00 |
| 211 | *CSK* | 0.3246 | 0.00 | 0.00 |
| 212 | *MAP3K5* | 0.3322 | 0.00 | 0.00 |
| 213 | *FGFR1* | 0.3396 | 0.00 | 0.00 |
| 214 | *RIPK3* | 0.3592 | 0.00 | 0.00 |
| 215 | *PLK3* | 0.3615 | 0.00 | 0.00 |
| 216 | *STK11* | 0.3687 | 0.00 | 0.00 |
| 217 | *SLK* | 0.3687 | 0.00 | 0.00 |
| 218 | *PDGFRBETA* | 0.3687 | 0.00 | 0.00 |
| 219 | *KIT* | 0.3687 | 0.00 | 0.00 |
| 220 | *MAP2K2* | 0.3757 | 0.00 | 0.00 |
| 221 | *ABL2* | 0.3757 | 0.00 | 0.00 |
| 222 | *RIPK1* | 0.3827 | 0.00 | 0.00 |
| 223 | *RIPK2* | 0.3965 | 0.00 | 0.00 |
| 224 | *MAP2K6* | 0.3965 | 0.00 | 0.00 |
| 225 | *PRKD2* | 0.4032 | 0.00 | 0.00 |
| 226 | *PRKCG* | 0.4165 | 0.00 | 0.00 |
| 227 | *PDPK1* | 0.4165 | 0.00 | 0.00 |
| 228 | *MAP3K14* | 0.4262 | 0.00 | 0.00 |
| 229 | *PKABETA* | 0.4359 | 0.00 | 0.00 |
| 230 | *FGR* | 0.4422 | 0.00 | 0.00 |
| 231 | *CK1DELTA* | 0.4607 | 0.00 | 0.00 |
| 232 | *IGF1R* | 0.4607 | 0.00 | 0.00 |
| 233 | *ABL* | 0.4786 | 0.00 | 0.00 |
| 234 | *AKT2* | 0.496 | 0.00 | 0.00 |
| 235 | *CAMKIIALPHA* | 0.496 | 0.00 | 0.00 |
| 236 | *PKD1* | 0.5016 | 0.00 | 0.00 |
| 237 | *MAP3K1* | 0.5038 | 0.00 | 0.00 |
| 238 | *PRKCQ* | 0.529 | 0.00 | 0.00 |
| 239 | *SYK* | 0.5548 | 0.00 | 0.00 |
| 240 | *ROCK1* | 0.5598 | 0.00 | 0.00 |
| 241 | *PKCGAMMA* | 0.5648 | 0.00 | 0.00 |
| 242 | *PKC* | 0.5978 | 0.00 | 0.00 |
| 243 | *PLK1* | 0.6102 | 0.00 | 0.00 |
| 244 | *MAP3K3* | 0.6232 | 0.00 | 0.00 |
| 245 | *MAPK9* | 0.6368 | 0.00 | 0.00 |
| 246 | *CAMK2A* | 0.7585 | 0.00 | 0.00 |
| 247 | *PKCALPHA* | 0.7745 | 0.00 | 0.00 |
